# Pareto optimality reveals an atlas of cellular archetypes

**DOI:** 10.1073/pnas.2530194123

**Published:** 2026-03-09

**Authors:** George Crowley, Uri Alon, Stephen R. Quake

**Affiliations:** ^a^Department of Bioengineering, Stanford University, Stanford, CA 94305; ^b^Department of Molecular Cell Biology, Weizmann Institute of Science, Rehovot 7610001, Israel; ^c^Department of Applied Physics, Stanford University, Stanford, CA 94305

**Keywords:** cell biology, biophysics, computational biology

## Abstract

We sought to identify universal organizing principles behind phenotypic variation within cell types. Pareto optimality describes how trade-offs between optimal solutions account for variation, predicting that the boundary points of a data distribution reflect specialized functions. We hypothesized that transcriptomic variation was explained by Pareto optimality across all cell types. We then used the Tabula Sapiens Atlas of single-cell RNA sequencing across cell types and tissues in the human body to test this hypothesis and found that most cell types adhere to this theory. This enabled us to use this principled method to characterize the functions performed by each cell type. These phenotypes are derived from an unbiased approach and do not incorporate ideas from existing biological models or theories, and yet in many cases they recapitulate our understanding of the functions of major cell types. Ultimately, we conclude that multiobjective optimization broadly shapes the observed phenotypic variation within cell types. This finding enables us to write explicit representations of the low-dimensional manifolds on which transcriptomes of single cells reside. This can inform the design of the next generation of virtual cell language models, which aim to statistically learn low-dimensional transcriptomic manifolds.

## Significance

Creating a first-principles molecular definition of cell type has been a challenging problem. We found that phenotypic variability within cell types is shaped by Pareto optimality, and therefore gene expression lies on low-dimensional polytopes (lines, triangles, tetrahedra, etc.). This approach provides a natural and unbiased definition of cellular archetypes and their functions without the need for prior biological knowledge.

Advances in single-cell RNA sequencing have made it possible to perform unbiased, high-throughput analysis of single-cell transcriptomes from entire organisms. Recently, the Tabula Sapiens Atlas has sequenced over one million cells across 28 organs and tissues of the human body, with replicates across donors (1). Similar projects have produced parallel databases for both human and a variety of model organisms (2–4), and substantial efforts have been made to compile these datasets (4–6). These atlases have allowed us to understand cellular heterogeneity across the entire human body, and their analysis has yielded rich insight across myriad cell types—both rare and common, including the immune (7–9) and stromal (10, 11) compartments. Major sources of variation within cell types have been discovered, including cell cycle phase, responses to extrinsic stimuli, epigenetic modulation, and bursty gene expression to name a few. Despite these advances, it is unclear if there are universal organizing principles that underlie these numerous sources of phenotypic variation within a given cell type. One natural place to look for such an organizing principle is multiobjective optimization. Pareto optimality is the baseline formulation of multiobjective optimization, and describes the situation where no explicit preference or weighting among the objectives is assumed. This concept is treated mathematically in the study of multiobjective optimization (12, 13), and separately applied to biology (14, 15).

We hypothesized that the phenotypic variation within cell types is explained by multiobjective optimization and used Tabula Sapiens to test this hypothesis. The Tabula Sapiens Atlas v1 is a single-cell RNA sequencing dataset containing 456,101 high-quality single cell transcriptomes processed via droplet microfluidic emulsion, covering 58,870 genes across 174 cell types, 25 tissues, and 15 donors (16). We applied quality control filters to remove outlier cells on several metrics, yielding 309,193 cells across 173 cell types, 24 tissues, and 14 donors, *SI Appendix*, Fig. S1 and Table S1. Cell type abundance filters left 110 cell types across the same number of tissues and donors, yielding 440 distinct donor-tissue-cell type strata for analysis (15, 17).

The only assumption we make in this analysis is that fitness is an increasing function of performance (14). Then, if there is a trade-off in performing multiple tasks, optimal phenotypes (i.e., those that maximize fitness) must lie in a region described by convex combinations of points that each maximize a single task’s performance (14). This region is called the Pareto front. *Any* pruning mechanism that removes nonoptimal phenotypes would restrict observed phenotypes to the Pareto front; pruning is a pervasive strategy across biology, and there could be a host of pruning mechanisms in multicellular organisms.

This approach does not require any assumptions about underlying regulatory dynamics or interactions among units. The Pareto front simply describes the region of optimal phenotypes, and its vertices are phenotypes each optimal at some task. Etiology and underlying regulatory dynamics can shape the Pareto front, but do not contradict that optimal phenotypes must lie on it (18). The elegance and power of Pareto optimality are that no specific selection mechanism or regulatory dynamics are required to arrive at its conclusions.

Phenotypes could fill the Pareto front or occupy subregions of it, depending on the form of the fitness function and externalities including but not limited to physical or biochemical constraints, or spatiotemporal variation in the environment (19, 20). In fact, Pareto optimality is compatible with and can organize discrete subtypes and continuous trajectories. Hence, only the two following requirements must be met to support that the variation in the data is explained by Pareto optimality: 1) phenotypes are bounded by a polytope in trait-space; and 2) the vertices of the polytope have enriched features (17). This conclusion can be further strengthened by identifying specific tasks associated with the enriched features, and showing the tasks’ recurrence across independent contexts (17).

Therefore, our analysis aimed to determine whether the Tabula Sapiens data are well described by polytopes, and whether the vertices of those polytopes have significantly enriched features. In finding that both conditions are indeed satisfied, we then found further support by showing that the vertices are functionally relevant based on our prior understanding of cell biology. These findings lead to the conclusion that multiobjective optimization broadly shapes phenotypic variation within cell types (17). This framework then allows us to infer which tasks of a given cell type may share a common biochemical environment.

## Most Cell Types Are Described by Polytopes.

**Fig. 1.**
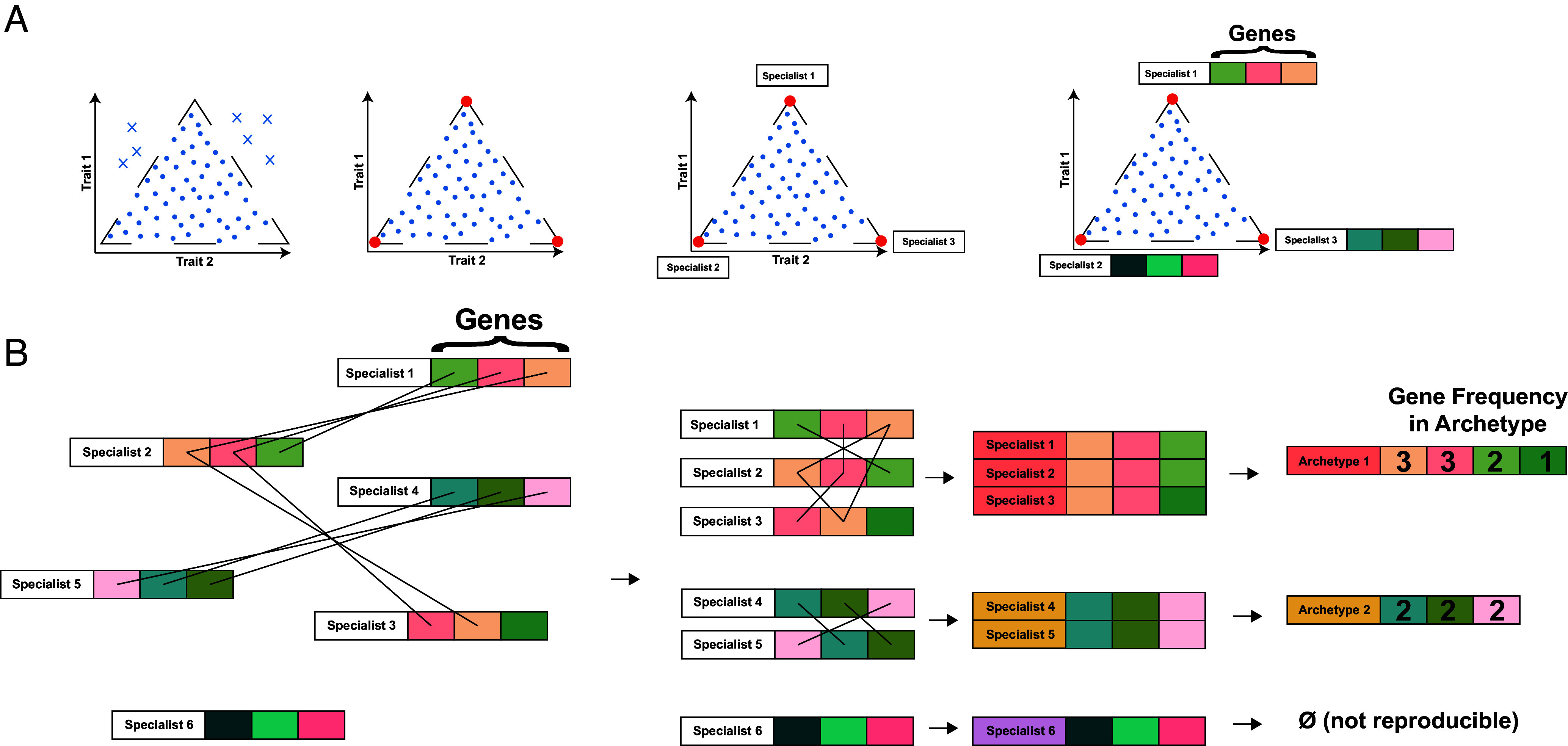
Process overview. (*A*) Pareto optimality theory. Pareto optimality places limits on allowable phenotypes. In trait space, this constrains phenotypes to be bound by a polytope. Specialist phenotypes lie at the vertices of the polytope and are enriched in traits relevant to their specialist functions. (*B*) Archetype Alignment. Vertices are clustered on a per-cell-type basis, yielding clusters that are filtered for reproducibility across donors and tissues.

We performed dimensional reduction and model fitting independently for each distinct donor-tissue-cell type dataset, Fig. 1*A*. We used this stratification to ensure that variation due to donor, tissue, or cell type did not define the space modeled, and instead only variation within a donor–tissue–cell type was captured. After removing mitochondrial genes and genes known to be affected by tissue processing, we normalized the remaining protein-coding genes to 10,000 counts (so that cell size effects would not dominate our results), and used principal component analysis (PCA) of protein-coding genes to represent cells in PCA space (16).

Several common steps in single-cell transcriptomics analysis are potentially incompatible with the analysis required to assess the dominance of Pareto optimality. Instead of selecting only highly variable genes, we retained all protein-coding genes for analysis because we did not want to introduce unknown bias into analyzed phenotypes, and it is also possible that non-highly-variable genes could still be enriched in cell populations near the archetypes.

Separately, data transformations are important to the space modeled in single cell transcriptomics. We only normalized the raw, protein-coding gene counts. Because the geometry of single-cell-type expression may be linear and the polytope vertices may be related to physical distances, we avoided common single-cell transformations that do not preserve pairwise distances, such as logging, scaling, and neighborhood-graph analysis (19). Finally, a density-based filter removed outliers in PCA space, stabilizing subsequent PCA transformations performed on linear-scale expression data (21). The use of this filter in archetypal analysis dates back over 30 y to the original archetypal analysis paper by Cutler and Breiman (22).

This experimental design precludes us from identifying rare cell populations by aggregating cells across donors or tissues. Thus, the density-based outlier filter is a conservative way to limit our analysis to the major cell populations, and try to achieve a more complete understanding of the variation of function just in these major populations. This is in-line with the goal of our investigation, which is to test if phenotypic variation within each cell type is broadly explained by multiobjective optimization.

Due to a previous suggestion in the literature that systematic technical artifacts could produce polytopal structures (23), we excluded artifactual genes that have previously been described to be affected by the tissue dissociation process (16) and mitochondrial genes (the 37 genes of the mitochondrial genome) before PCA to ensure they did not define the space. Following PCA, we dropped components that were correlated with artifactual or mitochondrial expression above a stringent threshold of |r| > 0.3. To further dispel concerns that polytopes may be related to technical artifact, these genes were still included in the enrichment analyses, and, as later elaborated, we observed no pattern of their enrichment at the vertices or influence on the fits. This gives us reason to believe that our observations in this space are driven by biological variation.

**Fig. 2.**
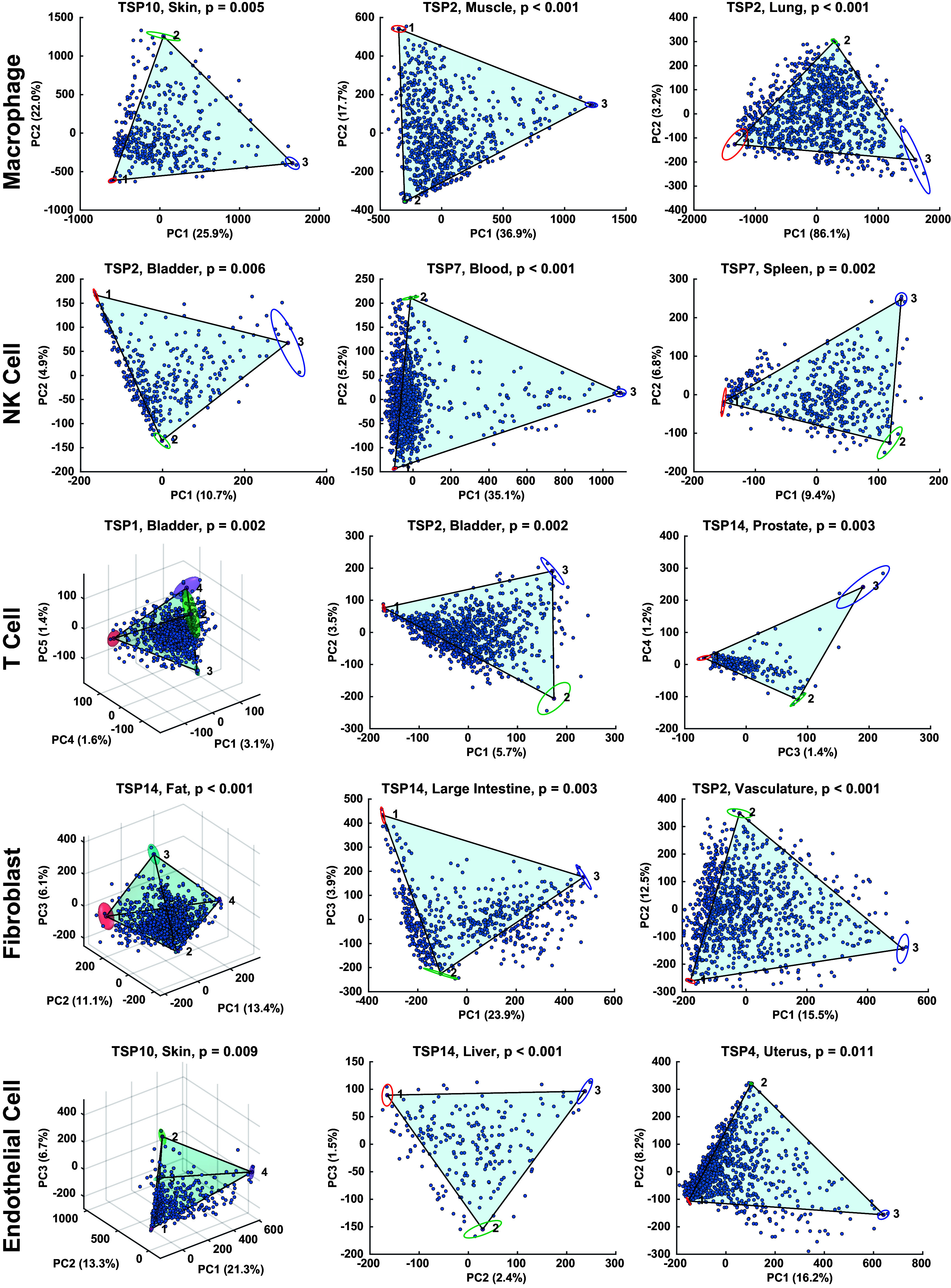
Reproducible geometry of donor-tissue-cell types and fits. Principal components analysis and Pareto Task Inference analysis performed independently on macrophages, NK cells, T cells, fibroblasts, and endothelial cells across 3 unique donor-tissues each demonstrates this geometry. Confidence bounds on vertex position are shown by ellipses and ellipsoids.

We performed polytope fitting on the single-cell transcriptomes of each donor-tissue-cell type, Fig. 2, with confidence bounds determined by bootstrapping, and *P*-values determined by the t-ratio test. The t-ratio measures the data’s similarity to a polytope (15, 17). The t-ratio test was performed by comparing the goodness of fits of shuffled and unshuffled data. The *P*-value was the proportion of times that the goodness of fit of shuffled data was better than that of the unshuffled data over 1,000 runs (17). To get properly calibrated *P*-values, the PCs are shuffled and not the genes. Hart et al. (17) provide an in-depth discussion of this test, and (23) shows it to be the more stringent of two procedures tested. We note that we specifically tested for simplices.

We set the significance threshold α = 0.05 for assessing significance of fit of a polytope to a given donor–tissue–cell type, and accepted a maximum false discovery rate (FDR) = 0.10 for determining which cell types were significantly fit overall. We labeled a cell type as significantly fit by polytopes overall if ≥50% of donors with that cell type available had significant tissues.

**Fig. 3.**
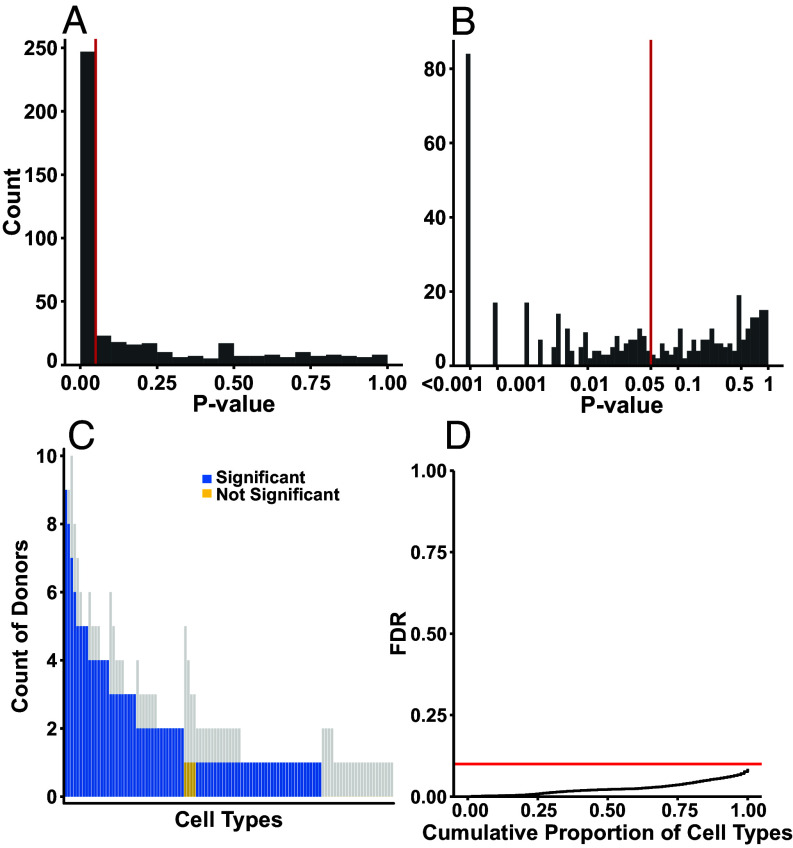
Most cell types are significantly well fitted by polytopes. Significance of fits of each donor-tissue-cell type in Tabula Sapiens [both (*A*) standard and (*B*) log-scaled axes shown]. (*C*) Counts of donors with significant tissue(s) present in each cell type, plotted against a background of all donors available for that cell type (gray background bars). Cell types with significance in at least 50% of available donors are considered significant. (*D*) Cumulative distribution function of FDR as a function of proportion of significant cell types retained. Three quarters of available cell types are significantly fit at α = 0.05 and maximum FDR = 0.10. Red lines are drawn at *P* = 0.05 (*A* and *B*) and FDR = 0.10 (*C*).

Three-quarters of cell types (82/110, 75%) were significantly well fitted by polytopes (defined by ≥50% of donors having significant tissues), Fig. 3 *A*–*C*. The associated FDR is 0.0849, Fig. 3*D*, which corresponds to roughly 7 false positives out of the 82 significantly fit cell types. Independently, a majority (247/440) of donor-tissue-cell type strata were each significantly fitted by polytopes. Furthermore, when removing singlet and doublet cell types (i.e., cell types with only one or two donor-tissues), 90% of cell types (35/39) were significantly fitted. We observed no substantial relationships between quality control metrics—including the percent of counts coming from artifactual genes—and significance of fits, *SI Appendix*, Fig. S2.

A detailed analysis of the frequency of significance of cell types shows that each donor and tissue has significantly fitted cell types, except for TSP3 (only a single donor-tissue-cell type available) and kidney (only 2 donor-tissue-cell types available), *SI Appendix*, Fig. S3. We have thus substantiated the first of the two requirements to show that the phenotypic variation is explained by Pareto optimality—the phenotypes are bounded by polytopes in trait-space.

**Fig. 4.**
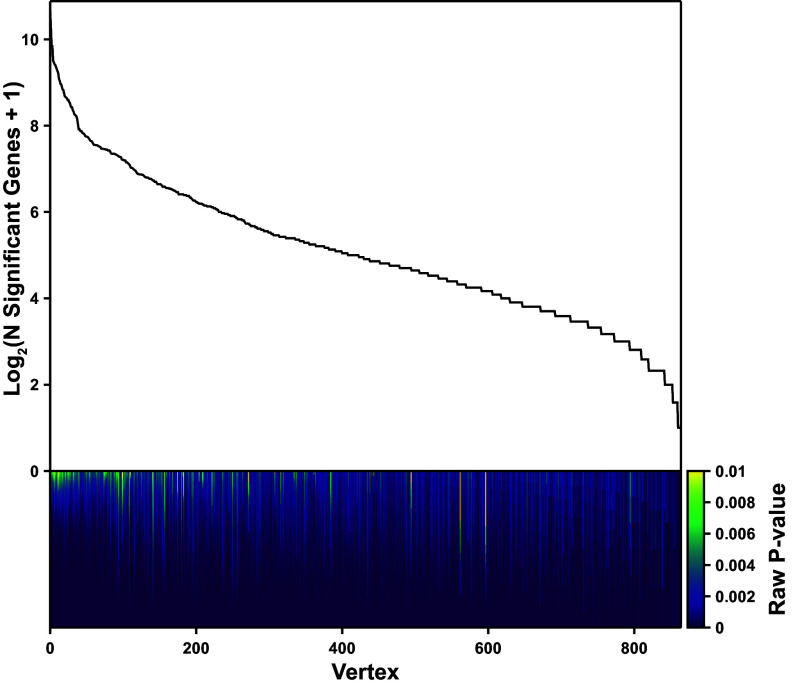
All vertices of significantly well fitted polytopes have significantly enriched genes. The number of significantly enriched genes in each of the 864 vertices from the 247 significant polytopal fits, plotted on log scale. Below, a stacked bar chart, with each segment representing a significantly enriched gene and colored by that gene’s *P*-value. Note that the range of the color bar goes from *P* = 0 to *P* = 0.01.

What remains is then to show that the vertices of these polytopes have enriched features. If the polytopes arose due to the structure of statistical noise present in the data, we would not expect to observe features that were significantly enriched in the cells nearest to the vertices (17). Across the 247 significant polytope fits of donor-tissue cell types, there were a total of 864 vertices, all of which had genes that were significantly enriched in the nearest cells as measured by Benjamini-Hochberg-corrected *P*-values with a maximum FDR of 0.10, Fig. 4. This dismisses the concern that the polytope data structure arose from statistical noise, and substantiates the second of the two requirements to show that the phenotypic variation is explained by Pareto optimality: the vertices of the phenotype-bounding polytopes have enriched features. At this point, all conditions have been satisfied to conclude that Pareto optimality explains the phenotypic variation of most cell types in the human body.

## Polytope Vertices Reflect Physiological Functions.

We next sought to identify specific tasks associated with the vertices and test whether those tasks recurred across independent contexts. Importantly, in Tabula Sapiens, there are 52 independent datasets (each donor-tissue that has significant cell types) across which to evaluate consistency.

The tissues with the highest number of donor-cell type pairs available were blood, spleen, and lung, *SI Appendix*, Fig. S4. The donors with the highest number of tissue-cell type pairs available were TSP14, TSP2, and TSP7, *SI Appendix*, Fig. S5. Finally, the cell types with the highest number of donor-tissue pairs available were macrophages, natural killer (NK) cells, T cells, fibroblasts, and endothelial cells, *SI Appendix*, Figs. S4 and S5. We therefore focused on these cell types across tissues to understand the correspondence of polytope vertices to biological functions.

Each of the 864 vertices potentially represents a biological function. In order to make headway, we focused on the most commonly observed vertices within the most abundant cell types. Thus, the remainder of our results focuses on broad strokes of the most confidently identified functions. There are many hundreds more vertices that could correspond to specialized cell functions that are tissue- or even donor-specific that we do not discuss here. We calculated gene enrichment at each vertex, and used the set of all protein-coding genes, including mitochondrial and artifact genes to allow the detection of artifactual vertices.

We restricted our functional inference to 670 vertices from the 35 cell types that were significantly fit overall and had more than two donor-tissues. Within each cell type, we then clustered vertices based on up to their 10 most highly differentially expressed genes, and further narrowed our focus to clusters that contained vertices from more than two-thirds of available donors for that cell type, with a correction factor to control false negatives. This set of filters is highly conservative and restricts us to identifying only the most common, broad strokes of cell function. Therefore, while there is rich subtype literature on cell types that we discuss, we focus on the dominating expression patterns per cell-type. It is worth noting that for each cell type there are many vertices that we do not plot or discuss that could correspond to niche functions. However, even given these restrictions, we will see below that this principled analytical approach still identifies universal cell functions that remain undercharacterized by current cell subtype schema.

For functional inference, we considered the genes that were shared among the vertices within a cluster. We used a large language model to provide a broad annotation for each gene list. Similar LLM approaches have been benchmarked recently and showed satisfactory agreement with gene ontology (24, 25). We used Gene Ontology-Biological Process, and several commercially available LLMs (*Materials and Methods*); Claude 4 Sonnet generally gave the most interpretable annotations. Note that these LLM-generated labels were not designed to provide detailed biological insight and we subsequently perform detailed manual comparison to established cell subtype repertoires.

## Archetypes Have Distinct Expressional Programs.

**Fig. 5.**
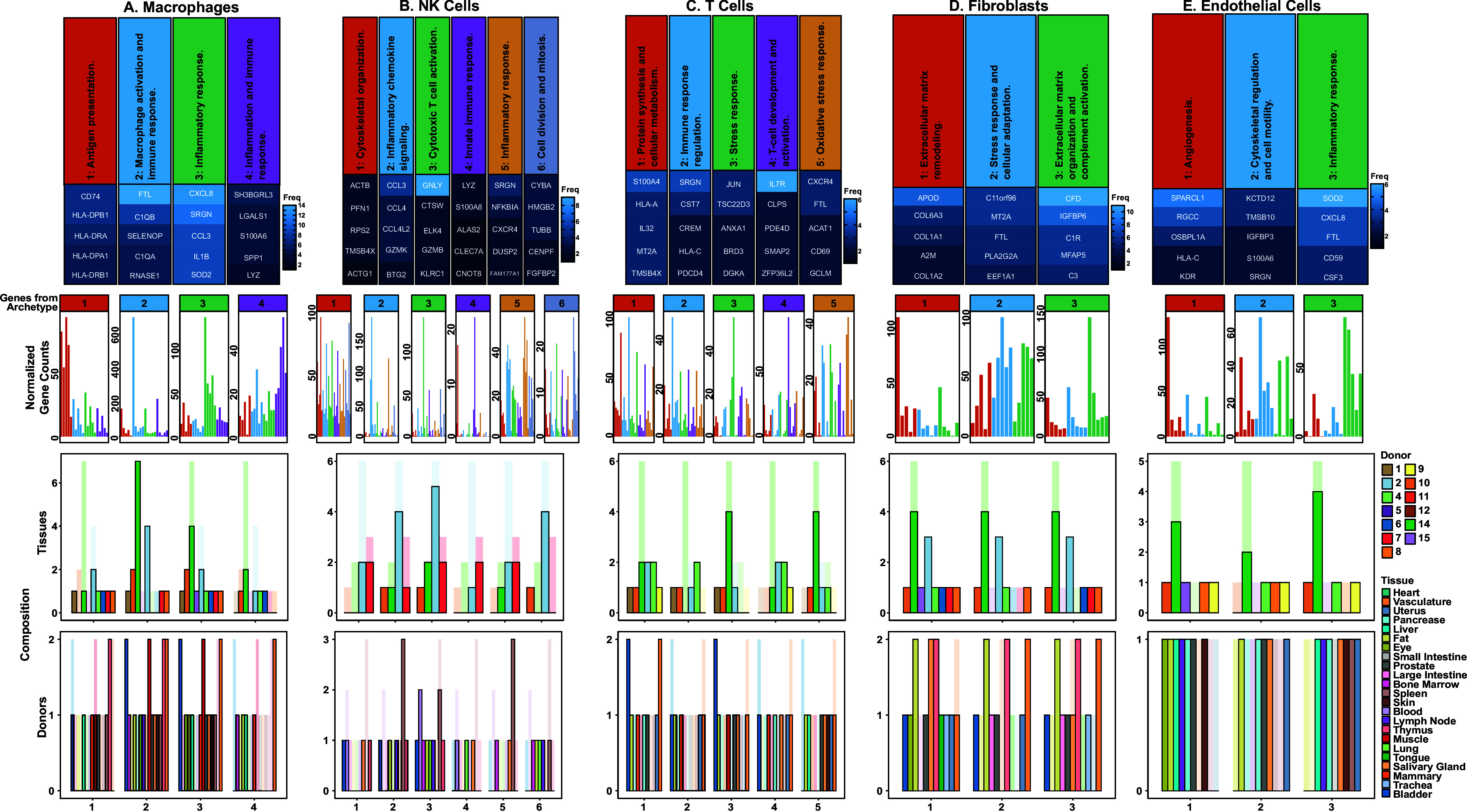
Archetypes of ubiquitous cell types. For 5 ubiquitous cell types [(*A*) Macrophages; (*B*) NK Cells; (*C*) T Cells; (*D*) Fibroblasts; and (*E*) Endothelial Cells], we show: 1st row: Heatmap of the top 5 most frequent archetype-defining genes in archetypes containing approximately two-thirds or more of available donors, annotated via a large language model; 2nd row: Gene expression of top 5 genes of each archetype, visualized across all archetypes, using the 5% of cells closest to each vertex on a donor-tissue-cell type basis. Within each archetype, genes are plotted in the descending order of appearance in the heatmap in the 1st row; 3rd row: Counts of tissues present in each donor for each archetype (opaque bars), plotted against a background of all tissues available for that donor-cell type (semitransparent bars); 4th row: Counts of donors present in each tissue for each archetype (opaque bars), plotted against a background of all donors available for that tissue–cell type (semitransparent bars).

We calculated the average gene expression of the defining genes of each archetype. We then compared the gene expression of archetype-defining genes within their own archetype to the expression of these genes in other archetypes. In all cell types discussed—macrophages, NK cells, T cells, fibroblasts, and endothelial cells—archetypes were defined by unique expressional programs; we conclude that the defining genes of each archetype are highly expressed within their own archetype, and lowly expressed in other archetypes, Fig. 5 *A*–*E* (second row). Thus, the archetypes are phenotypically distinct from each other.

## Macrophage Archetypes Reflect Immune, Metabolic, and Tissue Homeostatic Roles.

Macrophages are multitasking cells found in all tissues. Historic approaches subdivide macrophages into discrete categories such as M1 and M2 states. It has been argued that this taxonomy should be abandoned in favor of a function-based nomenclature (26). More recently macrophages have been divided into eight discrete substates, although data show that macrophage phenotype is continuous in gene expression space (8). Here, we applied a principled method to extract these functions without discretization and define them based on molecular expression. We find that macrophages are significantly fit by polytopes across tissues. Many of the archetype defining genes for macrophages have had specific tasks associated with them in prior literature. Here, we show how sets of these tasks may be consolidated under shared biochemical phenotypes.

The first macrophage archetype describes macrophages’ role in antigen presentation via the Major Histocompatibility Complex (MHC) class II pathway (with enrichment in *CD74*, *HLA-DRA*, *HLA-DPB1*, *HLA-DPA1*, *HLA-DRB1*, *HLA-DQA1*, *HLA-DRB5*), Fig. 5*A* (27). This archetype also shows macrophage roles in activation and amplification of the complement system (with enrichment in *C1QA*, *C1QB*, *C1QC*, *C3*) and homeostatic regulation of these processes (*CST3*) (28). Antigen presentation and complement activation are known to be expressed in macrophages across tissues and are key to core macrophage function and immunity (29). Our analysis indicates that these two functions may be performed by transcriptionally similar macrophage cells. This sheds light on an open question, which is to elucidate the transcriptional programs active in these pan-tissue functions, and assess the degree to which they are shared or unique (29).

The second macrophage archetype reflects macrophages specialized for long-term tissue residence and metabolic coordination (30). This population is characterized by iron homeostasis and storage (with enrichment in *FTL*, *FTH1*) (31), complement-mediated clearance functions (*C1QA*, *C1QB*, *C1QC*), (32) and antioxidant defense and tissue maintenance through Selenoprotein P (*SELENOP*) (33). Additional features include general RNA turnover and antimicrobial activity (*RNASE1*), cysteine protease inhibitors (*CSTB*, *CST3*), lipid metabolism regulation (*PLTP*, *APOE*, *APOC1*) (34), scavenger receptor-mediated clearance (*MARCO*, *MRC1*), and anti-inflammatory regulation (*VSIG4*) (35). This archetype represents tissue-resident macrophages that maintain tissue homeostasis through metal and lipid metabolic coordination and controlled immune surveillance.

The third macrophage archetype reflects macrophages’ role in the inflammatory response and was enriched in cytokine and chemokine production (*CXCL8*, *CCL3*, *IL1B*, *CCL4*, *CCL20*, *CCL3L1*, *CCL4L2*) and regulation (*SRGN*), as well as cell migration in response to inflammation (*GPR183*, also known as *EBI2*) (36). This population also shows metabolic adaptations for sustained inflammatory activity, including antioxidant defense during oxidative burst (*SOD2*) (37), energy metabolism to support high cytokine production (*NAMPT*), and metabolic reprogramming (*G0S2*) (38). Additionally, these macrophages exhibit early tissue repair signaling (*EREG*), indicating their role as sophisticated inflammatory coordinators that bridge innate immunity with tissue repair processes.

The fourth macrophage archetype represents highly motile macrophages specialized for acute inflammatory responses and tissue repair initiation. This population is distinguished by damage-associated molecular pattern (DAMP) and inflammatory signaling through S100 protein family members (*S100A4*, *S100A6*, *S100A8*, *S100A9*, *S100A10*) (39), matrix remodeling capacity (*SPP1*, *VCAN*, *VIM*), and enhanced cellular motility (*SH3BGRL3*, *PFN1*, *VIM*) (40). Additional characteristics include inflammation resolution programming (*LGALS1*), proteolytic activity (*CTSD*), and antimicrobial function (*LYZ*, *VIM*) (41). This archetype likely represents recruited macrophages derived from circulating monocytes that rapidly infiltrate inflamed tissues and coordinate both inflammatory responses and early tissue repair processes.

The first and second macrophage archetypes appear to share some functions, namely utilization of the complement system, its regulation, and antimicrobial activity. Indeed, Pareto optimality permits this manner of function sharing. Therefore, the defining function of the first archetype is antigen presentation through the MHC class II pathway, while the second archetype is distinguished by metabolic and tissue homeostatic functions. Complement (C1q) and cystatin (Cystatins B and C) production then seem to be performed without substantial preference by macrophages with either of these two specializations. In other words, antigen-presenting macrophages and those responsible for metabolic homeostasis seem to be similarly well-suited for the production of complement and cystatins.

Meanwhile, the fourth macrophage archetype expresses an additional form of antimicrobial activity via vimentin—a multipurpose protein also involved in cell motility and matrix remodeling. We note that while the fourth macrophage archetype shows an activated phenotype that would be consistent with extracellular secretion of vimentin, we cannot conclude vimentin’s specific fate from transcriptomics alone. However, it is perhaps efficient for this macrophage archetype to build several functions (antimicrobial activity, motility, and matrix remodeling) around the expression of one or more genes like *VIM* that are shared among these tasks. Finally, this archetype expresses *SH3BGRL3*, which encodes a protein that has been implicated in cell migration and TNF-α inhibition; This protein remains underexplored, and the present work highlights its importance to physiological macrophage function.

## Natural Killer Cell Archetypes Represented Motifs of Cytotoxicity and Immune Cell Recruitment.

NK cells are also found in all tissues and carry out cytotoxic functions. We found that NK cells are well described by a continuum of gene expression, Fig. 5*B*. The first NK cell archetype represents specialization in cytoskeletal organization and is characterized by expression of core cytoskeletal machinery including β-actin (*ACTB*), profilin-1 (*PFN1*), and thymosin β4 (*TMSB4X*). Together, these reflect cells specialized for dynamic actin polymerization and cytoskeletal remodeling essential for cell motility and target engagement. Profilin-1 regulates actin nucleation and elongation, while thymosin β4 sequesters actin monomers, thereby halting actin filament assembly (42). The presence of ribosomal protein S2 (*RPS2*) suggests active protein synthesis to support the high metabolic demands of cytoskeletal remodeling. Coordinated cytoskeletal dynamics are fundamental to NK cell function, enabling critical processes including tissue infiltration, granule secretion, immune synapse formation, and target cell conjugation (43). This archetype represents NK cells that have been optimized for coordinated cytoskeletal dynamics, thus recapitulating a crucial aspect of NK cell biology.

The second archetype demonstrates specialized inflammatory chemokine production through robust expression of C–C motif chemokine ligands (*CCL3*, *CCL4*, *CCL4L2*, *CCL3L1*). These cells also express granzyme K (*GZMK*), a unique granzyme that activates all components of the complement cascade (44). This combination suggests NK cells functioning as inflammatory coordinators that eliminate targets while simultaneously recruiting other immune effectors through potent chemokine production. The potential positive feedback between complement and macrophage inflammatory protein signaling lends further biological plausibility to this archetype. This archetype also expresses *CHMP1B*, which encodes a subunit of the ESCRT-III complex that is thought to contribute to granzyme and perforin resistance (45). Along with expression of BTG anti-proliferation factor 2 (*BTG2*) and heat shock protein A6 (*HSPA6*), this collection of genes could indicate a protective phenotype that allows NK cells to survive in challenging environments when initiating inflammatory signaling and cytotoxicity.

The third archetype represents highly cytotoxic NK cells expressing multiple killing mechanisms, including a potent mediator of cell death, granzyme B (*GZMB*), an antimicrobial peptide, granulysin (*GNLY*), and a cysteine protease, cathepsin W (*CTSW*). These cells express the inhibitory receptor NKG2A (*KLRC1*) and L-selectin (*SELL*), indicating mature NK cells with regulated cytotoxic capacity and tissue homing potential. The ELK4 transcription factor (*ELK4*) suggests active transcriptional programs supporting effector functions. This archetype embodies the classical NK cell cytotoxic program with perforin-granzyme-mediated killing complemented by granulysin’s antimicrobial and cytolytic activities.

The fourth archetype is distinguished by expression of lysozyme (*LYZ*) and S100 calcium-binding protein A8 (*S100A8*), indicating NK cells with enhanced antimicrobial functions. Lysozyme provides direct antimicrobial activity against bacterial cell walls, while S100A8 functions as a DAMP with additional antimicrobial properties. This minimal but functionally coherent gene signature suggests a specialized subset of NK cells that bridges traditional cytotoxic functions with antimicrobial immunity, potentially representing cells adapted for responses against intracellular pathogens or in inflammatory microenvironments where antimicrobial activity is prioritized.

The fifth archetype exhibits elevated *SRGN* for granule formation and degranulation, as well as *CXCR4* for tissue homing. Furthermore, this archetype expresses inflammatory response machinery, centered on transcriptional regulation through immediate early genes (*FOS*, *NR4A2*) and NF-κB signaling control (*NFKBIA*, *FAM177A1*), and MAPK downregulation (*DUSP2*). Meanwhile, *FAM177A1* is thought to attenuate the NF-κB cascade; however, its physiological roles—both broadly and specifically in NK cells—remain to be elucidated, and its pathological roles have only recently been studied (46). This archetype also expresses *SLA* (*SLAP*), which is thought to be involved in immune receptor signal transduction (47, 48). This archetype represents activated NK cells with transcriptional regulation that enables controlled inflammatory responses and cytokine production.

The sixth archetype captures proliferation through expression of key cell cycle machinery, including centromere protein F (*CENPF*) essential for kinetochore function, stathmin 1 (*STMN1*) for microtubule regulation, and tubulins (*TUBB*, *TUBA1B*) for mitotic spindle formation. These cells express cytochrome b-245 α (*CYBA*) for NADPH oxidase activity, high mobility group proteins (*HMGB2*, *HMGN2*) for chromatin remodeling and immune signaling, and fibroblast growth factor binding protein 2 (*FGFBP2*). The presence of granzyme B (*GZMB*) indicates maintained cytotoxic potential during proliferation. *FGFBP2* and *GZMB* are known marker genes of a previously described NK cell subtype: NK1/hNK_Bl1 cells (9, 49). Sphingosine-1-phosphate receptor 5 (*S1PR5*) suggests lymphoid tissue egress capacity. We also note that in addition to being a known modulator of inflammatory signaling, HMGN2 has a reported role in antimicrobial activity (50). Furthermore, microtubule regulation is important not just in the context of the cell cycle, but also for granzyme export. Therefore, this archetype represents expanding NK cell populations that retain effector functions while undergoing active cell division. These molecular signatures show that particular motifs of antimicrobial resistance and immune defense may be compatible with and efficiently consolidated under a mitotic phenotype.

These six NK cell populations reveal the sophisticated functional architecture underlying immune surveillance, from highly motile infiltrators to specialized killers to immune orchestrators. Recently, a comprehensive characterization of NK cells based on CITEseq and snRNAseq identified six subtypes of NK cells and aimed to standardize NK cell ontology. However, these subtypes were also acknowledged to be a continuum (9). The alignment of the archetypes with the subtypes identified by Rebuffet et al. supports the archetypes as biologically meaningful functional states rather than technical artifacts, and provides a natural resolution to the type vs. state distinction.

## Continuous Variation within T Cell Subtypes.

We identified five distinct T cell archetypes based on gene expression signatures, each representing functionally specialized immune states with unique biological roles and tissue adaptations, Fig. 5*C*. The first archetype represents metabolically active effector memory T cells with enhanced biosynthetic capacity. *S100A4* serves as a key marker of memory T cell status and is involved in cytoskeletal dynamics and migration (51). The signature includes genes for growth and proliferation regulation (*TPT1*), cytoskeletal regulation (*TMSB4X, *ACTB**), metal ion homeostasis (*MT2A*), and antigen presentation (*HLA-A*, *B2M*). *IL32* indicates pro-inflammatory cytokine production capability. This population likely represents tissue-resident effector memory T cells that maintain rapid response capabilities through enhanced metabolic activity (52).

The second archetype reflects T cells with balanced cytotoxic and regulatory functions. *SRGN* is associated with cytotoxic granule organization, while *CST7* provides protease inhibition to control cytotoxic responses. *CREM* acts as a transcriptional regulator of effector cytokines (53), and *PDCD4* functions as a translational repressor that can be modulated during activation. *HLA-C* contributes to antigen presentation capabilities and may influence interactions with NK cells or T cells through its unique binding properties compared to other MHC class I molecules. *RGCC* contributes to cell cycle control during proliferative responses. This archetype appears to represent cytotoxic T cells with built-in regulatory mechanisms to prevent excessive tissue damage while maintaining effector capabilities.

The third archetype defines T cells specialized for hostile microenvironments with enhanced stress tolerance. *TSC22D3* (*GILZ*) mediates glucocorticoid-induced anti-inflammatory responses, while *ANXA1* provides phospholipase A2 inhibition for anti-inflammatory activity. *JUN* contributes to AP-1 transcriptional responses, and the heat shock proteins (*DNAJA1*, *HSPA6*) provide protein quality control under stress conditions. *BRD3* broadly regulates inflammatory gene expression through histone binding and recruitment of transcriptional machinery, although its specific role in T cells remains unclear (54). This population likely represents tissue-resident T cells adapted to maintain function in challenging microenvironments while preventing excessive inflammatory responses (55).

The development and activation signature present in the fourth archetype characterizes transitional T cell populations maintaining developmental plasticity. *IL7R* expression indicates dependence on IL-7 for survival and homeostatic maintenance, typical of naive and memory precursor cells. *PDE4D* provides cAMP regulation during T cell activation and long-term responses (56). *ZFP36L2* offers posttranscriptional control by destabilizing cytokine transcripts (57). This archetype likely includes expression gradients in naive T cells transitioning to memory states, central memory T cell precursors, and potentially precursor exhausted T cells that retain proliferative and differentiation potential.

The fifth archetype represents T cell intratissue organization and management of oxidative stress during activation. *CD69* serves as a tissue residency marker that prevents lymph node homing, while *CXCR4* is broadly expressed across T cells (58) and provides responsiveness to CXCL12 spatial gradients in tissues. The antioxidant machinery includes *FTL* for iron sequestration, *ACAT1* for metabolic regulation, and *GCLM* for glutathione synthesis, which buffers ROS produced in activated T cells (59). This archetype captures continuous variation in *CXCR4* expression across the T cell population, and shows the metabolic machinery that enables T cell response.

Typically, T cells are divided in subtypes based on their place in developmental, activity, and functional hierarchies. The T cell archetypes capture core functions of T cells that vary within T cell subtypes, and are shared across them. We would thus interpret these functions as core to the T cell identity, and providing complementary information to the reported T cell subtypes (7). Archetype one provides rapid effector responses, archetype two balances cytotoxicity with regulation, archetype three maintains tissue protection under stress, archetype four preserves developmental plasticity, and archetype five manages oxidative stress during tissue-resident activation.

## Fibroblast Archetypes Highlight Distinct Roles in ECM Maintenance.

Fibroblasts are found in all tissues and have increasingly recognized regulatory roles beyond ECM building. We find three pan-tissue fibroblast archetypes that reflect roles in ECM maintenance, immune modulation, and environmental responsiveness, Fig. 5*D*. The first fibroblast archetype represents a specialized collagen production and matrix remodeling phenotype. This population is characterized by high expression of multiple collagen types (*COL1A1*, *COL1A2*, *COL3A1*, *COL4A2*, *COL6A2*, *COL6A3*), suggesting it functions as a collagen synthesis specialist. Beyond collagen production, this archetype shows matrix remodeling capabilities through matrix metalloproteinase expression (*MMP2*) and additional ECM components including laminin (*LAMB1*), and biglycan (*BGN*). The presence of growth factor binding proteins (*IGFBP7*) and matricellular proteins (*SPARCL1*, *FSTL1*) indicates this population not only produces structural ECM components but also creates signaling niches that modulate growth factor availability and cell–matrix interactions. Finally, *APOD* expression has been recently implicated in fibroblast dysfunction, although its specific role remains unclear (60, 61). This work implicates APOD in physiological fibroblast function, potentially related to supporting collagen synthesis or matrix remodeling through antioxidant properties.

The second archetype displays a robust cellular defense and metabolic adaptation phenotype. This population is distinguished by high expression of metallothioneins (*MT2A*, *MT1M*, *MT1X*), which protect against oxidative damage, heavy metal toxicity, and inflammatory insults. The coexpression of translational proteins (*RPL13*, *EEF1A1*) and stress-responsive transcription factors (*JUNB*) suggests elevated protein synthesis capacity alongside stress adaptation. This archetype likely represents fibroblasts primed for rapid response to environmental challenges while maintaining active protein production machinery.

The third archetype combines ECM structural functions with innate immune regulation. This archetype highly expresses complement factor D (*CFD*), which plays a dual role as both a complement system activator and an adipokine involved in metabolism and tissue repair. The expression of additional complement components (*C3*, *C1R*) and gelsolin (*GSN*) positions these fibroblasts as regulators of local immune responses. In addition, the expression of ECM organizing proteins including fibulin (*FBLN1*), decorin (*DCN*), and cystatin C (*CST3*) suggests these cells orchestrate ECM assembly while simultaneously modulating complement cascade activation, potentially serving as sentinel cells that integrate tissue homeostasis with immune surveillance. This archetype also expresses insulin-like growth factor binding protein 6 (*IGFBP6*), which is thought to play an important role in modulating matrix deposition following immune infiltration. However, the specific mechanisms remain to be fully elucidated (62, 63). The separation of collagen production from broader ECM organization and immune functions reflects that different aspects of connective tissue biology require distinct cellular specializations and biochemical environments.

## Endothelial Cell Archetypes Capture Their Roles in Tissue Homeostasis.

In our analysis of endothelial cell function, we identified three archetypes that reflect their roles in tissue homeostasis, repair, and protection, Fig. 5*E*. While labeled “angiogenesis” by an LLM, the first archetype represents a more complex collection of functions characteristic of capillary endothelial cells. This archetype is defined by *SPARCL1*, a matricellular protein originally discovered in and historically associated with high endothelial venules that regulates lymphocyte trafficking and vessel homeostasis (64). The archetype also expresses *RGCC*, a capillary endothelial cell marker (65) and a regulator of cell cycle that enables rapid remodeling capacity, and *KDR* (VEGFR2), indicating responsiveness to angiogenic signals. Additional components include *OSBPL1A*, a regulator of lipid metabolism and membrane organization, *NCOA7*, a regulator of metabolism, *S100A4*, a regulator of cytoskeletal dynamics and cell motility, and *HLA-C* for antigen presentation at the blood–tissue interface. This gene signature recapitulates the standard collection of functions performed by capillary endothelial cells (66, 67), adapted for monitoring local tissue conditions, facilitating selective immune cell trafficking, and maintaining vessel stability while retaining the capacity for rapid functional transitions. We also note that, because the arteriovenous transition represents a trajectory over space, this archetype captures a spatial coordinate of cell type organization within tissue.

The second endothelial cell archetype is defined by an enriched program focused on cytoskeletal regulation and cellular motility. The key markers of this archetype are β-thymosin family actin-sequestering proteins (*TMSB4X*, *TMSB10*) that play an important role in cytoskeletal organization by binding to and sequestering actin monomers, thereby inhibiting actin polymerization (68). Both thymosin-β4 and -10 have been implicated in the positive or negative regulation of VEGF expression, angiogenesis, and endothelial cell migration (69, 70). The expression of the cytoskeletal regulator and cell motility promoter *IGFBP3* reinforces this archetype’s specialization in dynamic cytoskeletal remodeling (71, 72). This archetype also expresses *KCTD12*, which encodes an auxiliary subunit of the GABA_B_ receptor (73). Despite its implication in cancer, little is known about the physiological function of KCTD12 (74, 75). Its presence in this archetype may support a cytoskeletal organizational role. Overall, this molecular signature indicates endothelial cells poised for rapid morphological changes, migration, and potentially involved in processes requiring extensive cellular reorganization such as sprouting angiogenesis, wound healing, or vascular remodeling.

The third archetype exhibits a comprehensive inflammatory response program centered around neutrophil activation and oxidative stress management. This archetype strongly expresses *CSF3* (*GCSF*), *CXCL8* (IL-8), and *SELE* (E-selectin), which coordinate neutrophil production, recruitment, and recognition of cytokine-activated endothelial cells, respectively. CXCL8 is one of the most potent neutrophilic chemokines and additionally promotes endothelial cell proliferation and migration during tissue repair through CXCR2 binding on endothelial cells (76). This archetype expresses a robust protective program against oxidative and inflammatory stress, including *SOD2* and metallothioneins (*MT1X*, *MT1E*, *MT2A*) for antioxidant and metal protection, *FTL* for iron sequestration, and *CD59*, a major inhibitor of complement-mediated cell lysis. *SOD2* encodes a superoxide dismutase that is essential for maintaining vascular function (77). This archetype represents endothelial cells activated for immune surveillance and inflammatory responses, capable of orchestrating neutrophil recruitment, managing oxidative stress, and coordinating tissue defense mechanisms.

These three endothelial archetypes represent distinct functional states that likely correspond to different vascular microenvironments and physiological demands. The angiogenesis-regulatory archetype maintains vessel homeostasis while retaining responsiveness to growth signals, the motility-specialized archetype facilitates dynamic vascular remodeling and repair, and the inflammatory-activated archetype manages immune responses and oxidative stress. This functional specialization allows the endothelium to adapt to diverse, tissue-specific requirements while maintaining its essential barrier and transport functions.

## Discussion

There are numerous methods to draw insight from continuous expression data, and many have been applied to single cell transcriptomic data. PCA, ICA, NMF, WGCNA, and topic modeling reveal coexpression modules (78), and trajectory inference methods, including Diffusion Pseudotime, reconstruct lineage trajectories, thus inferring dynamics (79, 80). However, *none* inherently ties their outputs to specific functional tasks. In contrast, Pareto optimality predicts that vertices of the data’s convex hull reflect distinct functions (12, 13, 15, 17). Archetypal analysis is then the natural choice to identify vertices on the hull (22). The advantages of archetypal analysis over SVD, PCA, ICA, NMF, and clustering have been clearly articulated in the literature, and we refer the reader to Mørup and Hanson 2012 for a succinct discussion on the matter (81). Note that archetypal analysis describes the data using a basis consisting of data points—in this case cells, not gene expression programs.

Our results indicate that we can answer the fundamental question posed at the beginning of this paper in the affirmative: The gene expression of most cell types is constrained to low-dimensional polytopes, the expression of key genes is significantly enriched at the vertices of those polytopes, and those genes correspond to functions performed by those cell types. Thus, we conclude that phenotypic variation within most cell types is explained by Pareto optimality. It directly follows that compositions of optimal cell states account for much of the observed intra-cell-type variation in the human body. Finally, we inferred which tasks of each cell type are efficiently performed in tandem, and which tasks may be divided between different states of that cell type. These results are robust as they are reproducible across donors and across different tissues with shared cell types. However, it is important to note that there are some limitations to the analysis and that not every cell type falls neatly into this paradigm, *SI Appendix*, Fig. S6.

First, there is clearly some experimental variation as for a given cell type, while a majority of donors may fit a polytope, some donors do not, and the source of this variation is unclear at this time. Also, a few cell types are not well fit by polytopes and it is unclear if this reflects their true biology or whether it is related to experimental artifacts. Nonetheless, it is notable that some of the most widely shared cell types across tissue are well described by polytopes and that the vertices can often be interpreted in terms of their biological function.

One possible source of artifacts in gene expression is tissue processing (16, 23). After attempting to control for this with several conservative filters, and ensuring reproducibility of our findings across donors and tissues, we did not, in general, observe artifactual genes appearing in our gene lists of interest. An exception might be the stress response archetype in fibroblasts, but we cannot say for certain that this phenotype is *only* present due to tissue processing artifacts; fibroblasts are known to be early responders to stress in healthy physiology.

In this study, we tested for simplicial structure, which is expected when the costs of performing tasks are additive and task execution is independent. When data fail this test, the underlying trade-offs may still be Pareto-like but with a different geometry—for example, a curved or nonconvex frontier, or a factorized structure (13, 14). This can come about if there are synergies between tasks, epistatic constraints, or discrete switching between cell states. Pareto structure can also be absent if there are no meaningful trade-offs. If a single task dominates, phenotypes should cluster near its optimum rather than lie on a trade-off boundary. More generally, if the system faced no meaningful constraints or competing objectives, there would be little reason to expect a Pareto geometry—though in practice it would seem unlikely that large populations of cells would be completely unconstrained.

Furthermore, the analysis could fail to detect polytopes due to statistical power (not enough cells), or if the data form a polytope in dimensions higher than those that we tested, and those polytopes’ lower dimensional projections were nonsimplicial. Another failure mode of Pareto analysis occurs when the fitted polytopes have no enriched features at their vertices. There are examples of how variance could produce such structures, and for this reason we would reject as a false positive any polytope with no features enriched at the vertices (17). In the present analysis, all vertices of all polytopes had enriched features, and so we did not reject any polytope due to this reason.

While we do not aim to elucidate the full expressional repertoires of each cell type, it may also be possible to increase the dimension of the polytope fits to uncover richer descriptions of cell repertoires. In the present analysis, we can draw conclusions about what might be the dominating tasks and how several tasks may be consolidated under a shared biochemical signature. Several transcripts discussed here have been previously implicated in niche-specific roles. Here, we implicate these genes as important mediators on a much broader scale. For example, SELENOP has previously been observed to be important to macrophages in muscle tissue, but this pan-tissue analysis supports a broader importance to tissue-resident macrophages. Many of the key macrophage genes that we discussed have previously been implicated in various inflammatory pathologies, especially cancer. In our analysis of nonpathologic tissues, we support these genes’ roles as key to general physiology. Additionally, we found several genes whose mechanisms and functions have not been fully elucidated, including *SH3BGRL3*, *FAM177A1*, *BRD3*, *APOD*, and *KCTD12*. This analysis allows us to speculate about their functional roles.

While we provide evidence for widespread Pareto constraints on gene expression, we have not identified the specific biological factors that generate the trade-offs. Still, it may be useful to sketch plausible sources of constraint and competition. Each cell type likely experiences a combination of general and type-specific constraints. General constraints could include limits on how many transcripts or proteins a cell can produce over a given time window, imposed by energy availability and expenditure, metabolic capacity, the kinetics of transcription and translation, the maximum number of accessible chromatin sites, or competition for resources at the cellular level. Alternatively, one can view the cell as operating under some fixed budget of transcripts—on the order of 10^5^ total mRNA molecules in a typical mammalian cell—that the cell must allocate optimally to accomplish some task(s), in concert with other cells of the same type.

Additional trade-offs may arise from cell type–specific regulatory and developmental mechanisms. For example, transcription factors could each impose their own constraints, similar to the well-studied case of sigma-factor competition in bacteria in heat shock protein production (14, 18). Another area to examine is processes that are described as competitive binding or inhibitory interactions. In multicellular systems, selection can also occur at the population level through contemporaneous pruning or competitive maturation processes, such as mechanisms implicated in B cell or T cell development, where cells may compete for limited survival or maturation cues. More broadly, quality-control or immune-surveillance processes could preferentially eliminate inefficient or maladaptive cells, thereby sharpening the observed trade-off structure at the population level.

Finally, Pareto optimality makes no statements about transitions or trajectories between states. In different contexts, optimal states can be terminal or not (see ref. 23 for an example of terminal differentiation at an archetype); the extent to which the same actual cells move between archetypes is still an open question (20, 82). In fact, a recent analysis modeled cells jointly by Pareto optimality and cell-circuit signaling loops to understand how regulatory dynamics impact differentiation (83).

In this context, regulatory dynamics—including bursting, feedback, and circuit-level interactions—can be viewed as candidate mechanistic implementations that populate the feasible region. Along with the forms of the performance and fitness functions, they can influence how densely different regions of the feasible set are occupied, and can carve out subregions that are rarely or never visited, without negating the presence of a Pareto bound. The specific pattern of occupation of the Pareto front (such as preferential occupation of the vertices, edges, or center, uniform occupation, or some other more complex pattern) could be biologically informative. Ultimately, we conclude that this principled method allows characterization of the collection of functions performed by a cell type.

## Conclusion

We used a principled method to identify archetypes that represent core functions performed by a cell type. For several ubiquitous cell types, we recapitulated known functions without incorporating prior biological knowledge. Placing these cellular tasks on a continuum, with cells performing some combination of specialized functions, advances our understanding of cell typology, beyond discretization of cell populations. Compared to canonical marker gene analysis, this continuous framing allows identification of functional marker genes that is unbiased, and captures continuous cellular heterogeneity. This approach provides a powerful way to combine two disjoint perspectives about cell type and cell state: whether they are discrete states or continuous states. The idea of having multiple discrete functions performed by a single cell type, and different cells within that type having a different balance of performing those functions that varies in a continuous fashion, provides a powerful paradigm to interpret and understand cell type and cell state distinctions.

## Materials and Methods

### Data Sources.

Single-cell RNA sequencing data were downloaded from public repositories. Sample preparation and processing has been previously described. Briefly, the single cell RNA sequencing dataset was previously processed including barcode-hopping correction, removal of cells expressing less than 200 genes, and removal of genes expressed in fewer than 3 cells. We also defined artifact genes as those potentially affected by tissue processing and dissociation (16).

### Data Preprocessing.

Additional preprocessing and filtering for the present study was performed in python 3.9 using Scanpy v1.10.3 (custom fork modified to add the intercept back to the results of scanpy.pp.regress_out(): https://github.com/ggit12/scanpy). We isolated droplet-microfluidic-emulsion-processed single cells for analysis. We removed genes expressed in fewer than 5 cells. We then followed a conservative approach to outlier removal and removed cells that were in the upper decile of any of: percent mitochondrial counts, percent artifact counts, number of genes expressed, total counts, and number of UMI counts. At this point, no cells had 100 counts or fewer of protein-coding genes (excluding mitochondrial and artifact genes, as this set of protein coding genes was eventually used to define the expressional phenotype in PCA space), and we mention this a-priori defined cutoff for completeness, *SI Appendix*, Fig. S1 and Table S1. This strategy allowed us to focus on major expressional trends and ignore edge cases. Mitochondrial genes and genes affected by tissue processing were then removed before normalizing the expression matrix to 10,000 counts/cell. On the filtered, normalized data, a cell cycling score was calculated as in ref. 16. The data were then stratified by cell type, donor, and tissue, and each stratum was analyzed separately, treating donors as biological replicates. Finally, as a conservative approach to outlier removal and to stabilize subsequent PCA transformations on linear-scale data, we removed cells in the bottom 10 percent of density in the first 3 components of PCA space for each donor–tissue–cell type (22).

### Data Analysis.

#### Pareto task inference analysis.

Gene expression data and cell metadata were then read into MATLAB R2022b for further processing using the Pareto Task Inference package (ParTI).

ParTI analysis was performed on all protein-coding genes as defined above, in all donor-tissue-cell types with at least 50 cells available for analysis after all filters in *data preprocessing*, which prior simulations have shown to be sufficient for polytope detection (23). Donor–tissue–cell types with more than 1,000 cells were downsampled to 1,000 cells. The ParTI algorithm was set to initialize with 5 dimensions and calculate the optimal number of vertices in the polytope based on an elbow-finding algorithm of the variance explained by successive archetypes (the default behavior). The ParTI package calculated *P*-values as the ratio of times that a polytope fit the real PCA data better than to randomly shuffled PCA data over 1,000 trials. When fitting polytopes, we swept a narrow range of dimensions (5 to 2 PCs), yielding between 6- and 2-vertex polytopes (specifically simplices), stopping when significance was observed at the optimal number of vertices for the given dimension. Prior works swept similar ranges (17, 23, 84), motivated by the following theory: The naïve expectation is that all the tasks of a system vary by orders of magnitude in terms of their impact on overall fitness, and only a few of the highest-magnitude tasks dominate phenotype space, with other tasks consolidated under generalist or specialist phenotypes (14). When visualizing fitted polytopes, vertices may appear nearby each other in low-dimensional projections. This can represent a simple projection artifact, or the splitting of a single specialist phenotype into biologically redundant ones.

The genes significantly enriched at each archetype (significant after the Benjamini-Hochberg multiple tests correction with a maximum FDR of 0.10) were then saved for archetype alignment. All genes, including mitochondrial and artifactual genes, were included in the enrichment analysis to allow detection of artifactual archetypes.

#### Statistical analysis.

To define the significance of a cell type, we set α = 0.05 and maximum FDR = 0.10. We then defined a cell type aggregation process, where a cell type was considered significant if 50% or more of available donors had a tissue with *P* < 0.05. For these cell types that qualified, we directly calculated the probability that the cell type was a false positive based on the number of donors and respective number of *P*-values calculated for each tissue of that donor (including excess *P*-values from testing multiple dimensions for significance), by assuming the false positive rate α of each donor-tissue-cell type, and applying a Bayesian correction factor for the empirical distribution of *P*-values (qvalue v2.30.0). This false positive probability per cell-type can then be used to directly calculate the FDR. Finally, all cell types up to a total FDR < 0.10 were considered well-fit by polytopes. This resulted in a total FDR of 0.0849 for the 82 significant cell types in this study (or about 7 false positives). Note that in Fig. 3*A*, we plot a single *P*-value per donor-tissue-cell type (the significant *P*-value if available, otherwise the first-calculated) to visualize the proportion of significant donor-tissue-cell types, while for statistical purposes and calculation of false discovery statistics, we used the full distribution of all *P*-values calculated, which is a conservative approach that may artificially overinflate the estimated FDR.

#### Quality control.

To ensure quality control of the fits, we represented quality control metrics in several ways. First, we plotted *P*-values of fits vs. each of the quality control metrics used in filter (except total counts protein-coding) and the total number of cells included in the fit, for all donor-tissue-cell types, and for donor-tissue-cell types from the five major cell types discussed in main, *SI Appendix*, Fig. S2. Then, to get a general assessment of the degree to which the quality control metrics may have affected the polytopal fits, we calculated the correlations of several metrics with the percent of cells that were within the bounds of the fitted polytope, *SI Appendix*, Table S2. Note that the main fitting algorithm (SDVMM) initialized with data points as vertices, and finds maximal polytopes that lie *within* the data by expansion, so it is expected that some cells are outside the bounds of the fitted polytope. Only very weak (|r| < 0.2) correlations were observed between any of the metrics we considered and the percent of cells that ended up within the final fit. In *SI Appendix*, Fig. S2 and Table S2, we used a single *P*-value per donor–tissue–cell type to attempt to detect quality control factors that may have impacted significant results; the same quality control analysis run on the full distribution of *P*-values showed smaller effect sizes.

#### Archetype alignment.

Our goal was to identify reproducible archetypes of each cell type. Therefore, we grouped all vertices by cell type, yielding several donor-tissues for each cell type, with each of these donor-tissues having multiple vertices. Each vertex represents a specialist phenotype that is associated with a ranked list of enriched genes. We used the number of shared genes between each specialist phenotype to align these phenotypes, Fig. 1*B*. This task was accomplished with the following graph-based approach. We defined an adjacency matrix for all specialist phenotypes found in a cell type based on the number of genes shared between each pair of specialist phenotypes. We then clustered (igraph v1.5.1) the specialist phenotypes according to the graph defined by this adjacency matrix, thereby aligning specialist phenotypes based on their shared genes. When at least 50% of biological replicates (i.e., donors) were represented within a cluster of specialist phenotypes, this cluster of specialist phenotypes was considered an archetype that represented a reproducible biological function of that cell type. This approach also elegantly handles the potential oversplitting of specialist phenotypes discussed above by aggregating biologically redundant specialist phenotypes from single donor-tissues. Then, redundant specialist phenotypes within a donor-tissue do not contribute to a cluster of specialist phenotypes meeting the donor-level reproducibility filter.

#### Archetype annotation.

To annotate each archetype based on the frequency-ranked list of genes shared across comprising specialist phenotypes, we first focused on the most frequent genes by dropping genes with frequency one in the archetype (i.e., present in only one specialist phenotype within that archetype). Then, we used a large language model (Claude 4 Sonnet) to annotate all archetypes across all cell types by passing the gene list of each archetype to the large language model and asking what biological process the gene list most likely represented. Notably, the large language model was *not* informed of the associated cell type, but, in several cases, still mentioned the correct cell type in the annotation. We accessed Claude 4 Sonnet through the Anthropic API with the R package claudeR v0.0.0.9000. This automation was needed to handle the large scale and breadth of this interpretative task. We tested several LLMs, including GPT-4, GPT-4o, Claude 3 and 4.1 Opus, and Claude 3.5 and 4 Sonnet. Results were generally similar across all the LLMs; we used Claude 4 Sonnet for final annotations as the Claude Sonnet series has been shown to be the most performant at gene set annotation (25).

#### Computational resources.

Computation for ParTI analysis was done on a high-performance computing cluster with a Slurm scheduler in Matlab R2022b. Downstream analysis was performed in R 4.2.1.

## Supplementary Material

Appendix 01 (PDF)

Dataset S01 (XLSX)

Dataset S02 (PDF)

## Data Availability

All source code required to reproduce this analysis is available in the pareto repository at https://github.com/ggit12/pareto and has been archived in Zenodo at https://doi.org/10.5281/zenodo.18766434 (85). Previously published data were used for this work (16). All other data are included in the manuscript and/or supporting information.

## References

[1] S. R. Quake, The Tabula Sapiens Consortium, Tabula Sapiens reveals transcription factor expression, senescence effects, and sex-specific features in cell types from 28 human organs and tissues. bioRxiv [Preprint] (2024). 10.1101/2024.12.03.626516 (Accessed 3 October 2025).

[2] H. Li , Fly cell atlas: A single-nucleus transcriptomic atlas of the adult fruit fly. Science **375**, eabk2432 (2022). 35239393 10.1126/science.abk2432 PMC8944923

[3] C. Tabula Muris, A single-cell transcriptomic atlas characterizes ageing tissues in the mouse. Nature **583**, 590–595 (2020). 32669714 10.1038/s41586-020-2496-1 PMC8240505

[4] A. Regev , The human cell Atlas. Elife **6**, e27041 (2017). 29206104 10.7554/eLife.27041 PMC5762154

[5] N. D. Youngblut , scBaseCount: An AI agent-curated, uniformly processed, and continually expanding single cell data repository (2025). https://doi.org:10.1101/2025.02.27.640494.

[6] S. Abdulla ; CZI Cell Science Program, CZ CELL×GENE Discover: A single-cell data platform for scalable exploration, analysis and modeling of aggregated data (2023). https://doi.org:10.1101/2023.10.30.563174. 10.1093/nar/gkae1142 PMC11701654 39607691

[7] C. Dominguez Conde , Cross-tissue immune cell analysis reveals tissue-specific features in humans. Science **376**, eabl5197 (2022). 35549406 10.1126/science.abl5197 PMC7612735

[8] K. Mulder , Cross-tissue single-cell landscape of human monocytes and macrophages in health and disease. Immunity **54**, 1883–1900.e1885 (2021). 34331874 10.1016/j.immuni.2021.07.007

[9] L. Rebuffet , High-dimensional single-cell analysis of human natural killer cell heterogeneity. Nat. Immunol. **25**, 1474–1488 (2024). 38956378 10.1038/s41590-024-01883-0 PMC11291291

[10] M. B. Buechler , Cross-tissue organization of the fibroblast lineage. Nature **593**, 575–579 (2021). 33981032 10.1038/s41586-021-03549-5

[11] S. N. Barnett , An organotypic atlas of human vascular cells. Nat. Med. **30**, 3468–3481 (2024). 39566559 10.1038/s41591-024-03376-x PMC11645277

[12] R. E. Steuer, Multiple Criteria Optimization: Theory, Computation, and Application (Wiley Series in Probability and Mathematical Statistics Applied Probability and Statistics, Wiley, New York, 1986), p. xx, 546 p.

[13] K. Miettinen, Nonlinear Multiobjective Optimization (International Series in Operations Research & Management Science, Kluwer Academic Publishers, Boston, 1999), p. xvii, 298 p.

[14] U. Alon, An Introduction to Systems Biology: Design Principles of Biological Circuits (CRC Press, Taylor & Francis Group, Boca Raton, ed. 2, 2020), p. xviii, 324 p.

[15] O. Shoval , Evolutionary trade-offs, Pareto optimality, and the geometry of phenotype space. Science **336**, 1157–1160 (2012). 22539553 10.1126/science.1217405

[16] C. Tabula Sapiens , The Tabula Sapiens: A multiple-organ, single-cell transcriptomic atlas of humans. Science **376**, eabl4896 (2022). 35549404 10.1126/science.abl4896 PMC9812260

[17] Y. Hart , Inferring biological tasks using Pareto analysis of high-dimensional data. Nat. Methods **12**, 233–235, 233 p following 235 (2015). 25622107 10.1038/nmeth.3254

[18] P. Szekely, H. Sheftel, A. Mayo, U. Alon, Evolutionary tradeoffs between economy and effectiveness in biological homeostasis systems. PLoS Comput. Biol. **9**, e1003163 (2013). 23950698 10.1371/journal.pcbi.1003163 PMC3738462

[19] M. Adler, Y. Korem Kohanim, A. Tendler, A. Mayo, U. Alon, Continuum of gene-expression profiles provides spatial division of labor within a differentiated cell type. Cell Syst. **8**, 43–52 e45 (2019). 30638811 10.1016/j.cels.2018.12.008

[20] M. Adler , Emergence of division of labor in tissues through cell interactions and spatial cues. Cell Rep. **42**, 112412 (2023). 37086403 10.1016/j.celrep.2023.112412 PMC10242439

[21] I. T. Jolliffe, Principal Component Analysis (Springer Series in Statistics, Springer, New York, ed. 2, 2002), p. xxix, 487 p.

[22] A. Cutler, L. Breiman, Archetypal analysis. Technometrics **36**, 338–347 (1994).

[23] Y. Korem , Geometry of the gene expression space of individual cells. PLoS Comput. Biol. **11**, e1004224 (2015). 26161936 10.1371/journal.pcbi.1004224 PMC4498931

[24] W. Hou, Z. Ji, Assessing GPT-4 for cell type annotation in single-cell RNA-seq analysis. Nat. Methods **21**, 1462–1465 (2024). 38528186 10.1038/s41592-024-02235-4 PMC11310073

[25] G. Crowley, Tabula Sapiens Consortium, S. R. Quake, Benchmarking cell type and gene set annotation by large language models with AnnDictionary. *Nat. Commun.* **16**, 9511 (2025). 10.1038/s41467-025-64511-x. PMC12569162 41152246

[26] M. Nahrendorf, F. K. Swirski, Abandoning M1/M2 for a network model of macrophage function. Circ. Res. **119**, 414–417 (2016). 27458196 10.1161/CIRCRESAHA.116.309194 PMC4965179

[27] C. Bleriot, S. Chakarov, F. Ginhoux, Determinants of resident tissue macrophage identity and function. Immunity **52**, 957–970 (2020). 32553181 10.1016/j.immuni.2020.05.014

[28] M. T. N. Tran , MafB is a critical regulator of complement component C1q. Nat. Commun. **8**, 1700 (2017). 29167450 10.1038/s41467-017-01711-0 PMC5700178

[29] E. Mass, F. Nimmerjahn, K. Kierdorf, A. Schlitzer, Tissue-specific macrophages: How they develop and choreograph tissue biology. Nat. Rev. Immunol. **23**, 563–579 (2023). 36922638 10.1038/s41577-023-00848-y PMC10017071

[30] L. C. Davies, S. J. Jenkins, J. E. Allen, P. R. Taylor, Tissue-resident macrophages. Nat. Immunol. **14**, 986–995 (2013). 24048120 10.1038/ni.2705 PMC4045180

[31] G. Mesquita , H-Ferritin is essential for macrophages’ capacity to store or detoxify exogenously added iron. Sci. Rep. **10**, 3061 (2020). 32080266 10.1038/s41598-020-59898-0 PMC7033252

[32] M. E. Benoit, E. V. Clarke, P. Morgado, D. A. Fraser, A. J. Tenner, Complement protein C1q directs macrophage polarization and limits inflammasome activity during the uptake of apoptotic cells. J. Immunol. **188**, 5682–5693 (2012). 22523386 10.4049/jimmunol.1103760 PMC3358549

[33] D. H. Hoang , Immune aging impairs muscle regeneration via macrophage-derived anti-oxidant selenoprotein P. EMBO Rep. **26**, 4153–4179 (2025). 40681871 10.1038/s44319-025-00516-3 PMC12373998

[34] A. Rouland , Role of apolipoprotein C1 in lipoprotein metabolism, atherosclerosis and diabetes: A systematic review. Cardiovasc. Diabetol. **21**, 272 (2022). 36471375 10.1186/s12933-022-01703-5 PMC9724408

[35] J. Li , VSIG4 inhibits proinflammatory macrophage activation by reprogramming mitochondrial pyruvate metabolism. Nat. Commun. **8**, 1322 (2017). 29109438 10.1038/s41467-017-01327-4 PMC5673889

[36] S. Hannedouche , Oxysterols direct immune cell migration via EBI2. Nature **475**, 524–527 (2011). 21796212 10.1038/nature10280 PMC4297623

[37] R. Rakkola, S. Matikainen, T. A. Nyman, Proteome analysis of human macrophages reveals the upregulation of manganese-containing superoxide dismutase after toll-like receptor activation. Proteomics **7**, 378–384 (2007). 17211829 10.1002/pmic.200600582

[38] B. L. Heckmann, X. Zhang, X. Xie, J. Liu, The G0/G1 switch gene 2 (G0S2): Regulating metabolism and beyond. Biochim. Biophys. Acta **1831**, 276–281 (2013). 23032787 10.1016/j.bbalip.2012.09.016 PMC3698047

[39] J. Austermann, C. Spiekermann, J. Roth, S100 proteins in rheumatic diseases. Nat. Rev. Rheumatol. **14**, 528–541 (2018). 30076385 10.1038/s41584-018-0058-9

[40] F. Di Pisa , SH3BGRL3 binds to myosin 1c in a calcium dependent manner and modulates migration in the MDA-MB-231 cell line. BMC Mol. Cell Biol. **22**, 41 (2021). 34380438 10.1186/s12860-021-00379-1 PMC8356473

[41] J. Arrindell, B. Desnues, Vimentin: From a cytoskeletal protein to a critical modulator of immune response and a target for infection. Front. Immunol. **14**, 1224352 (2023). 37475865 10.3389/fimmu.2023.1224352 PMC10354447

[42] B. Xue, C. Leyrat, J. M. Grimes, R. C. Robinson, Structural basis of thymosin-beta4/profilin exchange leading to actin filament polymerization. Proc. Natl. Acad. Sci. U.S.A. **111**, E4596–E4605 (2014). 25313062 10.1073/pnas.1412271111 PMC4217450

[43] A. Ben-Shmuel, B. Sabag, G. Biber, M. Barda-Saad, The role of the cytoskeleton in regulating the natural killer cell immune response in health and disease: From signaling dynamics to function. Front. Cell Dev. Biol. **9**, 609532 (2021). 33598461 10.3389/fcell.2021.609532 PMC7882700

[44] C. A. Donado , Granzyme K activates the entire complement cascade. Nature **641**, 211–221 (2025). 39914456 10.1038/s41586-025-08713-9 PMC12180478

[45] A. T. Ritter , ESCRT-mediated membrane repair protects tumor-derived cells against T cell attack. Science **376**, 377–382 (2022). 35446649 10.1126/science.abl3855

[46] J. N. Kohler ; Undiagnosed Diseases Network, Loss of function of FAM177A1, a Golgi complex localized protein, causes a novel neurodevelopmental disorder. Genet. Med. **26**, 101166 (2024). 38767059 10.1016/j.gim.2024.101166 PMC11451386

[47] J. U. Kazi, N. N. Kabir, L. Ronnstrand, Role of SRC-like adaptor protein (SLAP) in immune and malignant cell signaling. Cell. Mol. Life Sci. **72**, 2535–2544 (2015). 25772501 10.1007/s00018-015-1882-6 PMC11113356

[48] L. L. Dragone, L. A. Shaw, M. D. Myers, A. Weiss, SLAP, a regulator of immunoreceptor ubiquitination, signaling, and trafficking. Immunol. Rev. **232**, 218–228 (2009). 19909366 10.1111/j.1600-065X.2009.00827.x

[49] A. Crinier , High-dimensional single-cell analysis identifies organ-specific signatures and conserved NK cell subsets in humans and mice. Immunity **49**, 971–986.e975 (2018). 30413361 10.1016/j.immuni.2018.09.009 PMC6269138

[50] Y. Feng , Inhibitory effect of HMGN2 protein on human hepatitis B virus expression and replication in the HepG2.2.15 cell line. Antivir. Res. **81**, 277–282 (2009). 19150374 10.1016/j.antiviral.2008.12.011

[51] P. A. Szabo , Single-cell transcriptomics of human T cells reveals tissue and activation signatures in health and disease. Nat. Commun. **10**, 4706 (2019). 31624246 10.1038/s41467-019-12464-3 PMC6797728

[52] S. Ma, Y. Ming, J. Wu, G. Cui, Cellular metabolism regulates the differentiation and function of T-cell subsets. Cell. Mol. Immunol. **21**, 419–435 (2024). 38565887 10.1038/s41423-024-01148-8 PMC11061161

[53] S. H. Subramanyam, K. Tenbrock, The cAMP responsive element modulator (CREM) is a regulator of CD4(+) T cell function. Biol. Chem. **402**, 1591–1596 (2021). 34448385 10.1515/hsz-2021-0249

[54] N. Wang, R. Wu, D. Tang, R. Kang, The BET family in immunity and disease. Signal Transduct. Target. Ther. **6**, 23 (2021). 33462181 10.1038/s41392-020-00384-4 PMC7813845

[55] N. Asada , The integrated stress response pathway controls cytokine production in tissue-resident memory CD4(+) T cells. Nat. Immunol. **26**, 557–566 (2025). 40050432 10.1038/s41590-025-02105-x PMC11957990

[56] M. Bielenberg, R. Kurelic, S. Frantz, V. O. Nikolaev, A mini-review: Phosphodiesterases in charge to balance intracellular cAMP during T-cell activation. Front. Immunol. **15**, 1365484 (2024). 38524120 10.3389/fimmu.2024.1365484 PMC10957532

[57] F. Salerno , Translational repression of pre-formed cytokine-encoding mrna prevents chronic activation of memory T cells. Nat. Immunol. **19**, 828–837 (2018). 29988089 10.1038/s41590-018-0155-6 PMC6643272

[58] C. Cao , CXCR4 orchestrates the TOX-programmed exhausted phenotype of CD8(+) T cells via JAK2/STAT3 pathway. Cell Genom. **4**, 100659 (2024). 39317187 10.1016/j.xgen.2024.100659 PMC11602566

[59] T. W. Mak , Glutathione primes T cell metabolism for inflammation. Immunity **46**, 675–689 (2017). 28423341 10.1016/j.immuni.2017.03.019

[60] W. Xu , APOD acts on fibroblast-like synoviocyte and chondrocyte to alleviate the process of osteoarthritis in vitro. J. Orthop. Res. **42**, 296–305 (2024). 37728985 10.1002/jor.25690

[61] K. Takaya, T. Asou, K. Kishi, Identification of apolipoprotein D as a dermal fibroblast marker of human aging for development of skin rejuvenation therapy. Rejuvenation Res. **26**, 42–50 (2023). 36571249 10.1089/rej.2022.0056

[62] A. Liso , IGFBP-6: At the crossroads of immunity, tissue repair and fibrosis. Int. J. Mol. Sci. **23**, 4358 (2022). 35457175 10.3390/ijms23084358 PMC9030159

[63] L. L. Remsing Rix , IGF-binding proteins secreted by cancer-associated fibroblasts induce context-dependent drug sensitization of lung cancer cells. Sci. Signal. **15**, eabj5879 (2022). 35973030 10.1126/scisignal.abj5879 PMC9528501

[64] J. P. Girard, T. A. Springer, Modulation of endothelial cell adhesion by hevin, an acidic protein associated with high endothelial venules. J. Biol. Chem. **271**, 4511–4517 (1996). 8626806 10.1074/jbc.271.8.4511

[65] J. Kalucka , Single-cell transcriptome atlas of murine endothelial cells. Cell **180**, 764–779.e720 (2020). 32059779 10.1016/j.cell.2020.01.015

[66] M. Litvinukova , Cells of the adult human heart. Nature **588**, 466–472 (2020). 32971526 10.1038/s41586-020-2797-4 PMC7681775

[67] J. C. Schupp , Integrated single-cell atlas of endothelial cells of the human lung. Circulation **144**, 286–302 (2021). 34030460 10.1161/CIRCULATIONAHA.120.052318 PMC8300155

[68] F. X. Yu, S. C. Lin, M. Morrison-Bogorad, M. A. Atkinson, H. L. Yin, Thymosin beta 10 and thymosin beta 4 are both actin monomer sequestering proteins. J. Biol. Chem. **268**, 502–509 (1993). 8416954

[69] Y. Xing, Y. Ye, H. Zuo, Y. Li, Progress on the function and application of thymosin beta4. Front. Endocrinol. (Lausanne) **12**, 767785 (2021). 34992578 10.3389/fendo.2021.767785 PMC8724243

[70] H. Mu , Thymosin beta10 inhibits cell migration and capillary-like tube formation of human coronary artery endothelial cells. Cell Motil. Cytoskeleton **63**, 222–230 (2006). 16496302 10.1002/cm.20117

[71] R. Granata , Dual effects of IGFBP-3 on endothelial cell apoptosis and survival: Involvement of the sphingolipid signaling pathways. FASEB J. **18**, 1456–1458 (2004). 15247143 10.1096/fj.04-1618fje

[72] H. J. Lee, J. S. Lee, S. J. Hwang, H. Y. Lee, Insulin-like growth factor binding protein-3 inhibits cell adhesion via suppression of integrin beta4 expression. Oncotarget **6**, 15150–15163 (2015). 25945837 10.18632/oncotarget.3825 PMC4558142

[73] J. Schwenk , Native GABA(B) receptors are heteromultimers with a family of auxiliary subunits. Nature **465**, 231–235 (2010). 20400944 10.1038/nature08964

[74] Y. Suehara , KCTD12 is negatively regulated by Kit in gastrointestinal stromal tumors. Oncotarget **9**, 27016–27026 (2018). 29930747 10.18632/oncotarget.25469 PMC6007475

[75] Y. Zhong , KCTD12 promotes tumorigenesis by facilitating CDC25B/CDK1/Aurora A-dependent G2/M transition. Oncogene **36**, 6177–6189 (2017). 28869606 10.1038/onc.2017.287 PMC5671937

[76] S. Cambier, M. Gouwy, P. Proost, The chemokines CXCL8 and CXCL12: Molecular and functional properties, role in disease and efforts towards pharmacological intervention. Cell. Mol. Immunol. **20**, 217–251 (2023). 36725964 10.1038/s41423-023-00974-6 PMC9890491

[77] T. Fukai, M. Ushio-Fukai, Superoxide dismutases: Role in redox signaling, vascular function, and diseases. Antioxid. Redox Signal. **15**, 1583–1606 (2011). 21473702 10.1089/ars.2011.3999 PMC3151424

[78] W. Saelens, R. Cannoodt, Y. Saeys, A comprehensive evaluation of module detection methods for gene expression data. Nat. Commun. **9**, 1090 (2018). 29545622 10.1038/s41467-018-03424-4 PMC5854612

[79] L. Haghverdi, M. Buttner, F. A. Wolf, F. Buettner, F. J. Theis, Diffusion pseudotime robustly reconstructs lineage branching. Nat. Methods **13**, 845–848 (2016). 27571553 10.1038/nmeth.3971

[80] W. Saelens, R. Cannoodt, H. Todorov, Y. Saeys, A comparison of single-cell trajectory inference methods. Nat. Biotechnol. **37**, 547–554 (2019). 30936559 10.1038/s41587-019-0071-9

[81] M. Mørup, L. K. Hansen, Archetypal analysis for machine learning and data mining. Neurocomputing **80**, 54–63 (2012).

[82] D. P. Cook, J. L. Wrana, A specialist-generalist framework for epithelial-mesenchymal plasticity in cancer. Trends Cancer **8**, 358–368 (2022). 35183479 10.1016/j.trecan.2022.01.014

[83] S. Miyara , Cold and hot fibrosis define clinically distinct cardiac pathologies. Cell Syst. **16**, 101198 (2025). 39970910 10.1016/j.cels.2025.101198 PMC11922821

[84] A. Nath , Evolution of core archetypal phenotypes in progressive high grade serous ovarian cancer. Nat. Commun. **12**, 3039 (2021). 34031395 10.1038/s41467-021-23171-3 PMC8144406

[85] G. Crowley, U. Alon, S. R. Quake, Pareto. Zenodo. 10.5281/zenodo.18766434. Deposited 24 February 2026.

