# Supplementary material for: Pareto optimality reveals an atlas of cellular archetypes
Source: Proc Natl Acad Sci U S A. 2026 Mar 9;123(11):e2530194123. doi: 10.1073/pnas.2530194123 (PMC12993957; doi:10.1073/pnas.2530194123)
Supplement: Supplementary file 1 — Appendix 01 (PDF) [file pnas.2530194123.sapp.pdf]

PNAS

Supporting Information for
Pareto Optimality Reveals an Atlas of Cellular Archetypes

George Crowley[1], Uri Alon[2], Stephen R. Quake[1,3] *
Corresponding author: steve@quake-lab.org

The PDF file includes:

Figures S1 to S6
Tables S1 to S2

Other Supplementary Materials for this manuscript include the following:

Dataset S1
Dataset S2

Supplemental Figures

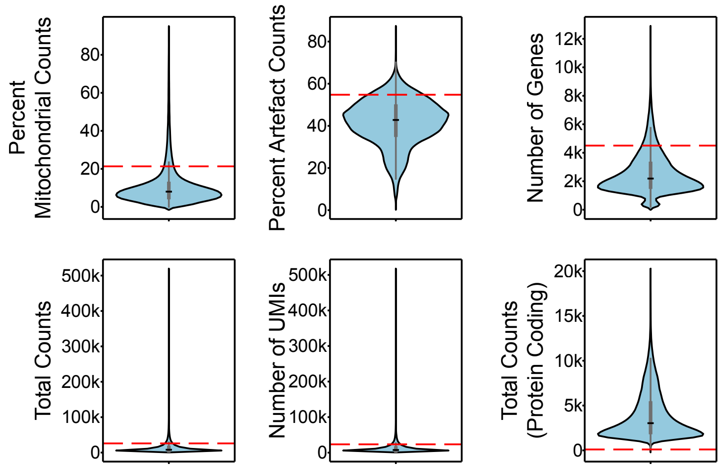

Figure S1. Distribution of percent mitochondrial genes, number of genes expressed, total counts, and number of UMI counts across cells. Vertical line indicates 90th percentile.

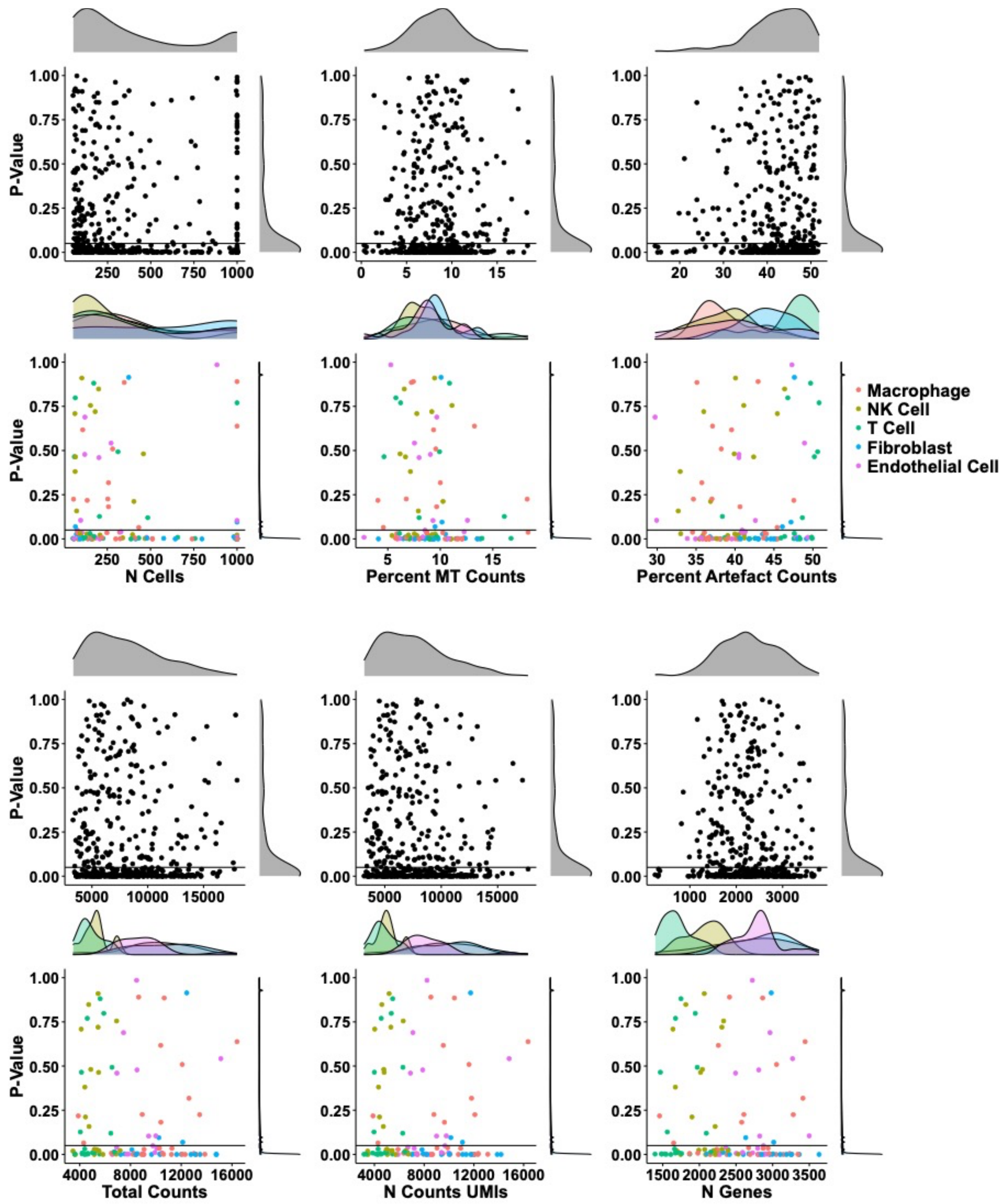

Figure S2. Scatterplots of p-value of polytopal fit vs. quality control metrics for all donor-tissue-cell types (greyscale) and for the five major cell types discussed in main (colored by cell type).

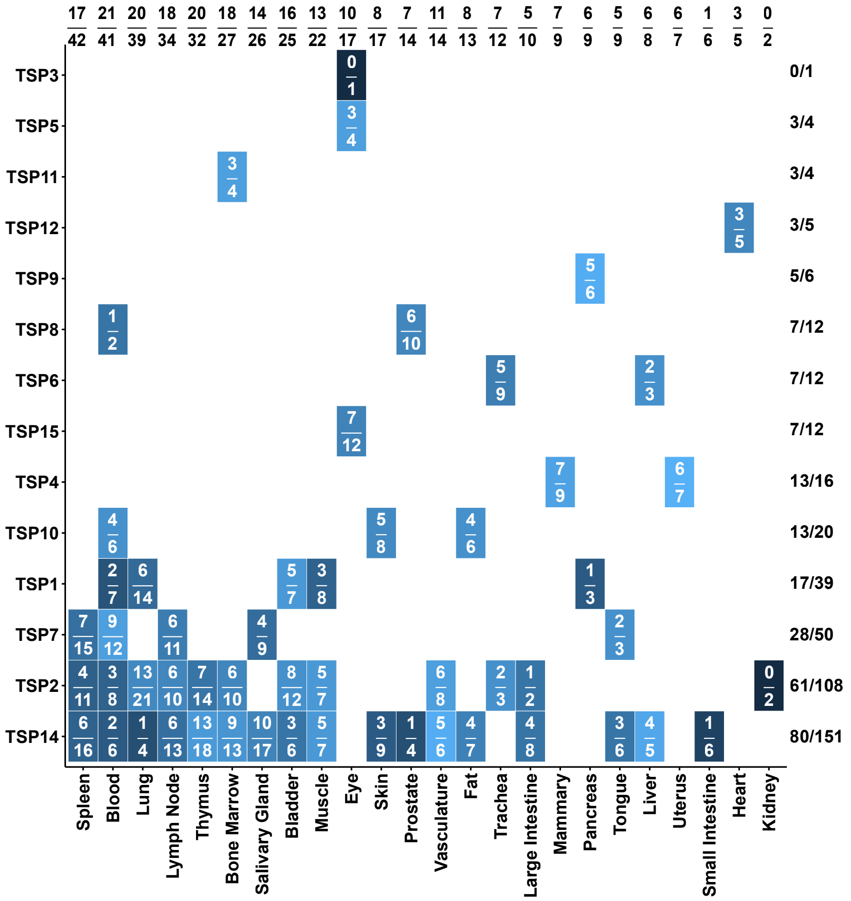

Figure S3. Donor-by-tissue heatmap of significant p-values, labeled with number of significant cell types over number of available cell types for each tile in the heatmap. The heatmap has marginal row and column sums of number significant over number available.

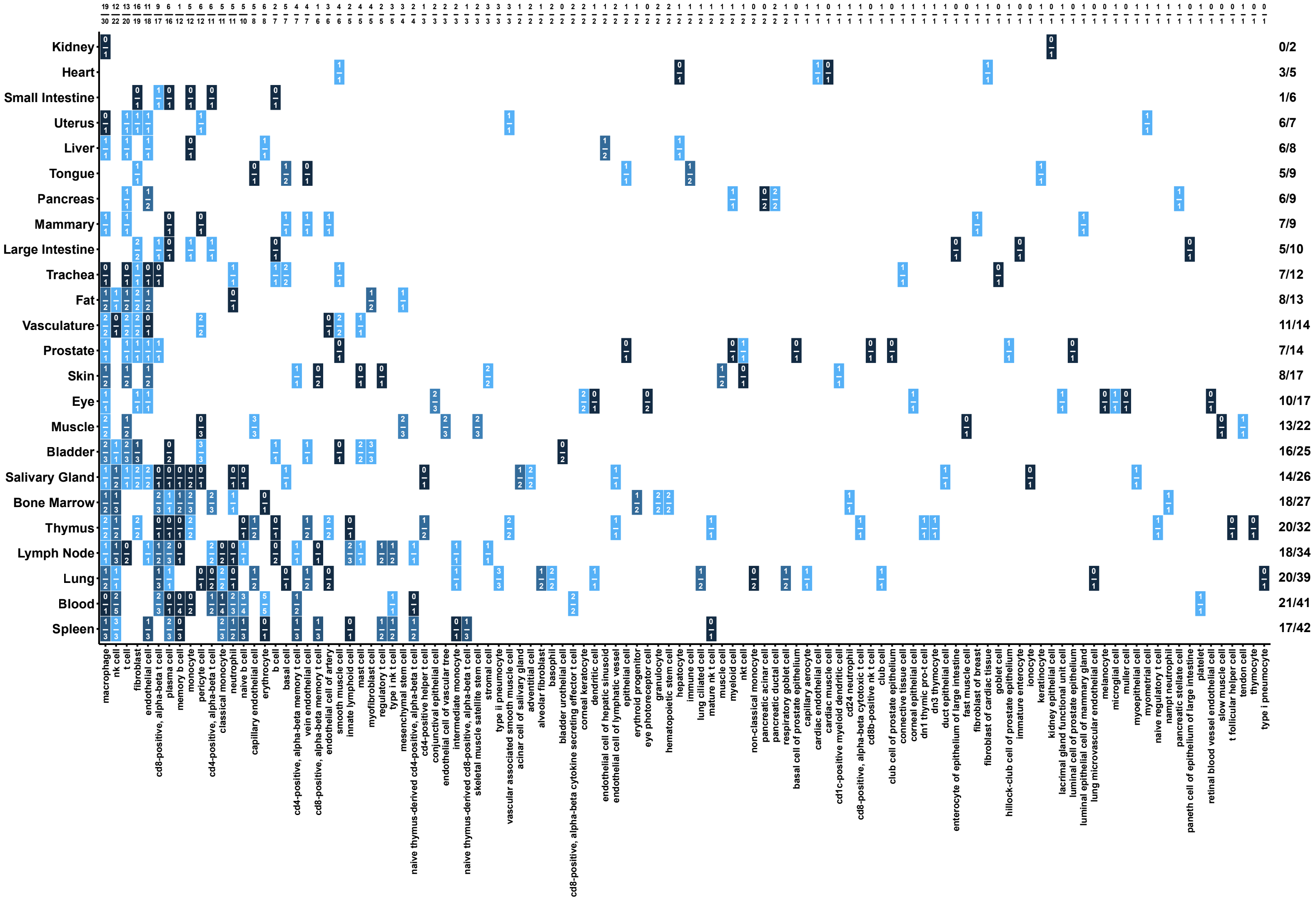

Figure S4. Tissue-by-cell type heatmap of significant p-values, labeled with number of significant donors over number of available donors for each tile in the heatmap. The heatmap has marginal row and column sums of number significant over number available.

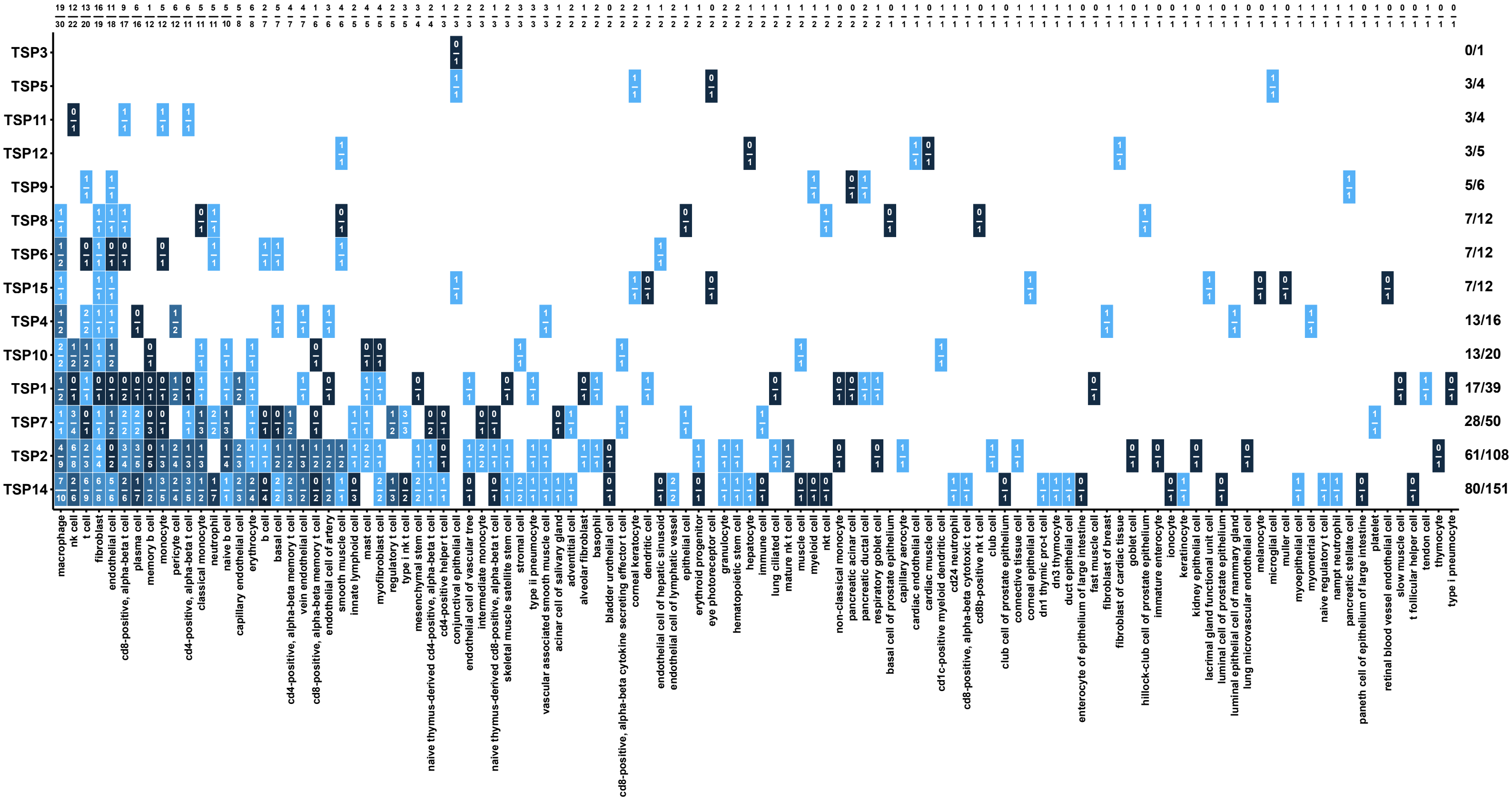

Figure S5. Donor-by-cell type heatmap of significant p-values, labeled with number of significant tissues over number of available tissues for each tile in the heatmap. The heatmap has marginal row and column sums of number significant over number available.

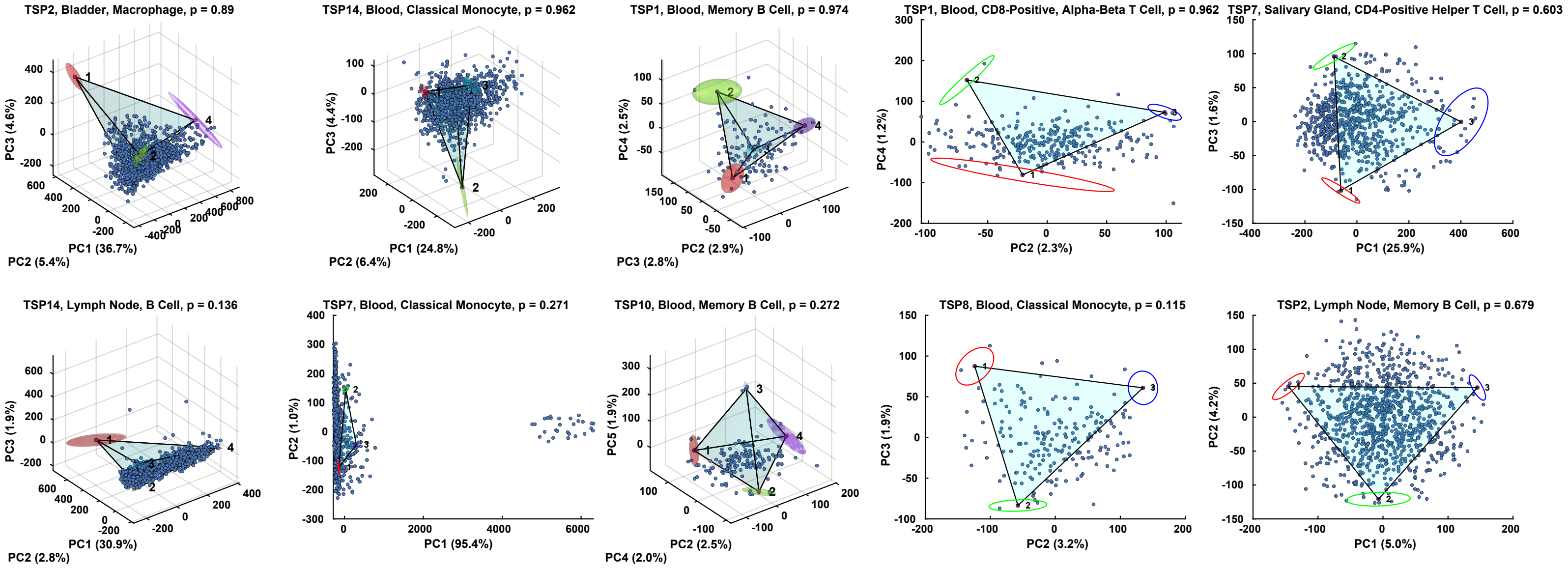

Figure S6. Not all donor-tissue-cell types are fit by simplices. Principal components analysis and Pareto Task Inference analysis where fits were not significant show a variety of failure modes. Confidence bounds on vertex position are shown by ellipses and ellipsoids.

Supplemental Tables

Quality Control Metric	Threshold Value	N Cells Excluded	Bound
Percent Mitochondrial Counts	21	45611	Upper
Percent Artefact Counts	55	45611	Upper
Number of Genes	4503	45636	Upper
Total Counts	25921	45612	Upper
Number of UMIs	23424	45617	Upper
Total Protein-coding Counts	100	0	Lower
Threshold values rounded to nearest integer.			

Table S1. Quality control thresholds used in initial filtering of Tabula Sapiens and number of cells/genes beyond the bound of each threshold. All thresholds except "Total Protein-coding Counts" were based on the 90th percentile of the data.

Percent of Cells in Polytope vs.	R	P-Value
P-value < 0.05	0.19	0
Mean(Percent Counts Artefact)	-0.12	0.01
Mean(Total Counts)	0.1	0.04
Mean(N Counts UMIs)	0.09	0.07
Mean FC(N Genes)	-0.06	0.21
Mean(Percent Counts MT)	-0.05	0.29
Mean FC(N Genes by Counts)	-0.03	0.59
Mean FC(Percent Counts MT)	-0.02	0.62
Mean FC(N Counts UMIs)	-0.02	0.74
Mean(N Genes by Counts)	0.02	0.66
N Cells	-0.02	0.63
Mean FC(Percent Counts Artefact)	0.01	0.88
Mean FC(Total Counts)	0	0.93
Mean(N Genes)	0	0.92
Mean Fold Change (Mean FC)		

Table S2. Correlations of quality control metrics with the percent of cells inside the fitted polytope.

Supplemental Files

Dataset S1 Significantly enriched genes for all cell types and archetypes that passed filters described in main. Two sheets included: 1) Top_5_Cell_Types: information for the 5 cell types discussed in main based on having the most donor-tissues; and 2) All_Cell_Types: information for all cell types.

Dataset S2. An Atlas of Cellular Archetypes. Archetype figures (same form as **Figure 5**) for all 35 cell types, including cell types not discussed in main manuscript.
